# Applications of CRISPR/Cas tools in improving stress tolerance in *Brassica* crops

**DOI:** 10.3389/fpls.2025.1616526

**Published:** 2025-09-02

**Authors:** Linh Bao Ton, Zuhra Qayyum, Junrey Amas, William J. W. Thomas, David Edwards, Jacqueline Batley, Aria Dolatabadian

**Affiliations:** ^1^ School of Biological Sciences, The University of Western Australia, Perth, WA, Australia; ^2^ The Institute of Agriculture, The University of Western Australia, Perth, WA, Australia; ^3^ Centre for Applied Bioinformatics, The University of Western Australia, Perth, WA, Australia

**Keywords:** Brassica, CRiSPR/Cas, genome editing, disease resistance, stress, transformation, resistance genes, plant breeding

## Abstract

*Brassica* species, which include economically important *Brassica* crops grown around the globe, are important as popular vegetables, forage, and oilseed crops, supplying food for humans and animals. Despite their importance, these crops face increasing challenges from biotic and abiotic stresses, exacerbated by climate change and the evolving threat of crop pathogens. Enhancing crop resilience against these stresses has become a key priority to ensure stable crop production. Recent advancements in genomic studies on *Brassica* crops and their pathogens have facilitated the deployment of CRISPR/Cas systems in breeding major *Brassica* crops. This review highlights recent progress in CRISPR/Cas-based gene editing technologies to improve resistance to pathogens and enhance tolerance to drought, salinity, and extreme temperatures. It also summarises the molecular mechanisms underlying crop responses to these stresses. Furthermore, the review discusses the workflow for employing the CRISPR/Cas system to boost stress tolerance and resistance, outlines the associated challenges, and explores prospects based on gene editing research in *Brassica* species.

## Graphical Abstract

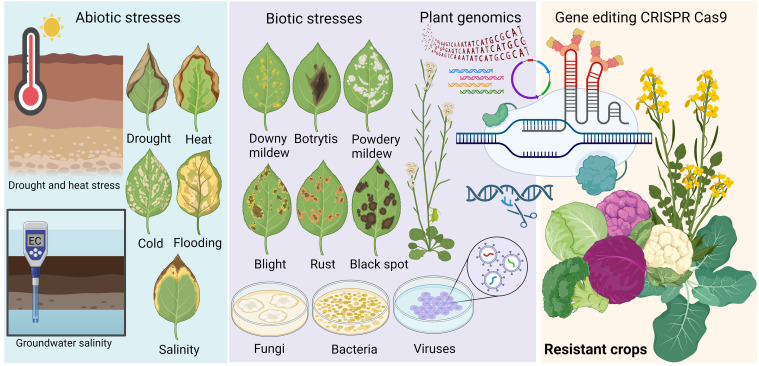

## 1 Introduction

The *Brassica* genus in the Brassicaceae family comprises around 159 species, many of which are of economic importance, providing seed oil, condiments, and vegetables (Bhattacharya, 2019; Borges et al., 2023). *Brassica* vegetables are cultivated and consumed worldwide, involving different cultivars of cabbage, cauliflower, broccoli, brussels sprouts (*Brassica oleracea* var. *gemmifera*), and kale (Podsędek, 2007). Meanwhile, *B. napus* (canola) is one of the most important oilseed crops planted globally and is the leading crop amongst other *Brassica* oilseed crops such as winter turnip rape (*B. rapa* subsp. *oleifera*), swede *(B. napus* spp. *napobrassica)*, Indian mustard (*B. juncea*), Ethiopian mustard (*B. carinata*) and black mustard (*B. nigra*) cultivated in selected regions of the world (Kirkegaard et al., 2021). The genomic relationships of these *Brassica* species described by U, N, (1935) comprises three diploid and three allopolyploid species, including *B. oleracea* (CC, 2*n* = 18), *B. rapa* (AA genome, 2*n* = 20), *B. nigra* (BB, 2*n* = 16), *B. carinata* (BBCC, 2*n* = 34), *B. juncea* (AABB, 2*n* = 36), and *B. napus* (AACC, 2*n* = 38).

Both oilseed and vegetable *Brassica* species play important roles in global food security, with a steady increase in their production since 2000 (FAOSTAT, 2023). *Brassica* vegetables are good sources of vitamins and antioxidants and protect against certain cancers and inflammation (Francisco et al., 2017). According to FAOSTAT (2023), cauliflower and broccoli are among the world’s primary crops, with a total production of approximately 26 million tonnes (MT) in 2022. Based on the average production of cauliflower and broccoli during the 1994–2022 period, China was the leading producer with around 7.5 MT, followed by India (6.3 MT), the US (1.1 MT), Spain (0.5 MT) and Italy (0.4 MT). China is the leading cabbage producer, with 29.9 MT, followed by India (7.0 MT) and Russia (2.9 MT) (FAOSTAT, 2023). *Brassica* vegetable production plays an important economic and social role in developing countries, such as African countries. In Kenya, cabbage is cultivated on small to medium-scale farms and is an important crop for tackling nutrition poverty due to its highly nutritious components (Daniel et al., 2023). *Brassica* oil crops, such as *B. napus* and *B. juncea*, provide canola oil for multiple industries, such as food, feed, fertiliser, and biodiesel. Canola oil is the third most consumed oil after soybean and palm oil (Adwiyah et al., 2023; USDA, 2023). Recently, *Brassica carinata* has been cultivated as a rotated crop for biofuel production (Basili and Rossi, 2018). Demand for vegetable and oilseed *Brassica* species is forecast to increase in the next decade due to the increasing global population. Besides, recent changes in renewable energy policies in some countries have led to the expansion of the biofuel market (OECD-FAO, 2023) and allowed the use of canola oil in biofuel production, boosting canola oil demand. However, global *Brassica* crop production faces challenges, such as limitations in arable land and consequences of climate change, such as the emergence of more virulent plant pathogens and more intensive abiotic stresses. Diseases caused by microbial pathogens can cause up to 90% of yield losses in *Brassica* species (Greer et al., 2021), while heat stress and UV-B radiation cause significant yield reduction (Secchi et al., 2023; Sehgal et al., 2022).

Breeding for disease resistance and abiotic stress tolerance in *Brassica* crops has been dramatically improved with advancements in genomics (Boivin et al., 2004; Das et al., 2024) and genome editing tools (Bao et al., 2019; Chen et al., 2019; Bhadauria et al., 2024). Nonetheless, the adverse consequences of climate change continue to challenge *Brassica* crop yield and have been linked to the emergence of novel strains of plant microbial pathogens that overcome current resistance in cultivated crops (Borges et al., 2023; Guerret et al., 2017; Van de Wouw et al., 2014). Fortunately, recent advancements in functional genomics of *Brassica* crops have revealed complex genetic networks and molecular mechanisms underlying their response to abiotic and biotic stressors, from which key genes and genomic factors controlling the response have been identified, allowing for the intervention of novel gene editing technologies (Wu et al., 2020; Varanda et al., 2021; Tan et al., 2024; Wang et al., 2024). Since 2012, the **c**lustered **r**egularly **i**nterspaced **s**hort **p**alindromic **r**epeats/CRISPR-associated protein (CRISPR/Cas) systems modified from the bacterial adaptive immune system of bacteria and archaea (Jinek et al., 2012) have been the most widely used genome editing tool worldwide (Talakayala et al., 2022; Wada et al., 2022). Compared to genome editing nucleases, such as zinc-finger nucleases (ZFNs) (Carroll, 2008; Cathomen and Keith, 2008) and transcription activation-like (TAL) effector nucleases (TALENs) (Christian et al., 2012; Li and Yang, 2013), RNA-directed CRISPR-Cas systems are more popular due to their versatility, simplicity, and feasibility (Li et al., 2025; Guo et al., 2023; Nerkar et al., 2022). Briefly, a typical CRISPR/Cas system for genome editing involves two main components a single Cas effector protein with one or two nuclease domain(s) and one or multiple CRISPR RNA (crRNA) sequences, with an additional trans-activating CRISPR RNA (tracrRNA) in case of the Cas9 systems (Charpentier et al., 2015; Jiang and Doudna, 2017). Each crRNA contains a spacer (30–40 bp) at the 5’ end, complementary to a sequence of a foreign DNA source, and a CRISPR repeat (25–35 bp) at the 3’ end (Jiang and Doudna, 2017). For the Cas9 and most Cas12 systems, the base pairing of the repeat sequence of crRNA with tracrRNA forms guide RNA (gRNA). The gRNA directs the Cas protein to a complementary DNA site (~20 nucleotides), or DNA targets flanked by specific protospacer adjacent motifs (PAMs) (Asmamaw and Zawdie, 2021). For the Cas9 and Cas12 systems, Cas protein in complex with gRNA cleaves the double-stranded DNA (dsDNA) target and generates a double-stranded break (DSB). Modifications (e.g. indels, base editing) are introduced at DSBs through natural cellular DNA repair pathways called non-homologous end joining (NHEJ), where the ends of cleaved DNA are re-joined, and homologous recombination (HR), where the DNA gaps are synthesised based on the homologous template (Cathomen and Keith, 2008; Li and Heyer, 2008). Therefore, Cas9 and Cas12 systems are powerful tools for plant functional genomics and improving crop traits (Nerkar et al., 2022; Hussain et al., 2018). In the RNA targeting systems, such as CRISPR/Cas13a-d, Cas13 protein is directed to its target mainly by gRNA and is less dependent on PAMs for target recognition, of which the Cas13c and Cas13d system has no PAM preference (Mahas et al., 2019; Cox et al., 2017). The single-stranded RNA target is cleaved upon being bound by the CRISPR/Cas13 (Cox et al., 2017). Hence, the CRISPR/Cas13 has been quickly adopted for targeting RNA viruses to improve plant resistance to viruses and diagnose viral diseases (Hagit et al., 2024; Zhan et al., 2023; Shihong Gao et al., 2021).

Over the past two decades, CRISPR/Cas-based precise genome modification has rapidly evolved in its application in plant crops, including *Brassica* species (Li et al., 2024). Benefiting from the *Brassica* omics findings, the CRISPR/Cas tools further characterise functions of candidate genes and key genetic elements regulating plant resilience traits (Amas et al., 2023; Zhang et al., 2023), enable precise mutation induction in the *Brassica* genomes that conferring resistance/tolerance to biotic and abiotic stress. This review highlights the recent progress in deploying CRISPR/Cas tools in *Brassica* crops, focusing on resistance to microbial pathogens and tolerance to major abiotic stresses, such as heat, drought, and salinity which reveal potential genes controlling these processes were summarised in **Table 1**. The workflow for utilising the endonuclease system is also discussed.

**Table 1:** CRISPR/Cas application for addressing biotic and abiotic stresses in Brassica.

Stressors	Targeted gene	Targeted genome	Cas enzyme/type of modification	Improved phenotypes	References
*S. sclerotiorum resistance*	*BnaA03.MKK5, BnaA06.MPK3/BnaCO3.MPK3*,	*B. napus*	Cas9/overexpression	Enhanced hypersensitive response cell death	Zhang et al., 2021
	*BnaA03.WRKY28*/*BnaA09.VQ12*	*B. napus*	Cas9/knock-out	Enhanced hypersensitive response cell death	Zhang et al., 2022
*S. clerotiorum* and *Botrytis cinera* resistance	Receptor like kinase (BnaA05.RLK-902)	*B. napus*		Enhanced resistance to the two pathogens.	Zhao et al., 2024
*P. brassicae* resistance	Bna-APS4 (a sulphate adenyl-transferase gene)	*B. rapa*	Cas9/knock-out	Enhanced resistance to clubroot disease	Zhou et al., 2024
Fusarium wilt and Xanthomonas black rot resistance	*BoBPM6* and *BoDMR6* genes	*B. oleracea*	Cas9/knock-out	Improved clubroot and black rot resistance	Zhang et al., 2025a
Black rot resistance	*BoSWEET15b*	*B. oleracea var. captitata L.*	Cas9/base editing	Improved black rot resistance	Kaur et al., 2023a
TuMV resistance	*eIF(iso)4E genes (Bra035531, Bra039484, and Bra035393)*	*B. rapa*	Cas9/indel	Improve resistance to TuMV	Lee et al., 2023a
	*eIF(iso)4E.c*	*B. rapa*	Cas9/frameshift	Increased jasmonic acid content and TuMV resistance	Liu et al., 2024
CMV	CMV ss-RNA genome	*A. thaliana*	FnCas9/viral RNA silencing	CMV resistance	Zhang et al., 2019
TRV	TRV genome	*A. thaliana*	LshCas13a /viral RNA cleaving	TRV resistance	Aman et al., 2018a
Abiotic stress	*OPEN STOMATA 2 (OST2)*	*A. thaliana*	Cas9/frameshift	Increased stomatal response	Osakabe et al., 2016
Drought	*vacuolar H+ pyrophosphate (AVP1) regulatory gene*	*A. thaliana*	Cas9/upregulation	Improved drought stress tolerance	Park et al., 2017b
	*abscisic acid-responsive element binding (AREB1)*	*A. thaliana*	dCas9/histone acetylation	Enhanced drought stress response	Roca Paixão et al., 2019
Drought	*trehalase 1 (TRE1)*	*A. thaliana*	Cas9/indel	Increased trehalose accumulation and cell viaability	Nuñez-Muñoz et al., 2021
Drought	*BnaA6.RGA*	*B. napus*	Cas9/frameshift	Improved drought tolerance	Wu et al., 2020
ER stress	*elongated hypocotyl 5 (HY5)*	*B. rapa*	Cas9/indel	Improved drought tolerance	Lee et al., 2023b
Drought	*B. napus nuclear factor YA7 (BnaA9.NF-YA7)*	*B. napus*	Cas9/knockout	Increased drought tolerance, reduced stomatal conductance and transpiration rate.	Wang et al., 2024
Drought and high salt	*ABA-induced transcription repressor (AITR) family genes*	*A. thaliana*	Cas9/knockout	enhanced drought and salinity tolerance	Wang et al., 2015; Chen et al., 2021
Toxic metal	*BnaNRAMP1*	*B. napus*	Cas9/knockout	Reduced Cd accumulation	Zhang et al., 2025b
Toxic metal	*BnCUP1*	*B.napus*	Cas9/knockout	Reduced Cd accumulation	Yao et al., 2022

## 2 Improvement of biotic stress tolerance in *Brassica*

To cope with pathogen infection, plants have developed two layers of immune systems. The first layer, pathogen-associated molecular patterns (PAMPs) triggered immunity (PTI), is activated by surface-localised pattern recognition receptors (PRRs) upon recognising (PAMPs), microbe/damage-associated molecular patterns (MAMPs and DAMPs) (Iqbal et al., 2021; Ngou et al., 2022). The second layer is effector-triggered immunity (ETI), which is induced by recognition of pathogen-secreted effector proteins by plant resistance proteins. The resistance proteins are usually receptors consisting of nucleotide-binding leucine rich repeat (NLR) domains (Dalio et al., 2021). Most identified resistance genes mentioned below are NLRs.

### 2.1 Alternatives for enhancing resistance to fungal diseases

The fungal pathogens *Alternaria* spp.*, Fusarium oxysporum, Leptosphaeria maculans*, and *Sclerotinia sclerotiorum* are the primary pathogens affecting *Brassica* crop yield worldwide (Zheng et al., 2020a). To overcome plant host PTI, fungi produce specialised virulence factors called effector proteins that interact with plant host resistance proteins encoded by (*R*) genes, activating ETI (Tsuda and Katagiri, 2010; Spoel and Dong, 2012);. Resistance (*R)* genes are the key components of the plant immune response involved in pathogen recognition and the activation of the defence response (Chisholm et al., 2006).

As an efficient system to generate mutants, CRISPR/Cas9 has contributed to recent developments in the knowledge of plant-fungal pathogen interactions, unveiling the intricate dynamics of these processes. This ribonucleoprotein (RNP) complex has been utilised to eludcidate plant response mechanisms to to identify and confirm the functions of major host and fungal factors involved in the interactions. Chitin, a major component of fungal cell walls, functions as a PAMP in plants and animals (Liu et al., 2020). Host plants counteract fungal invasions by degrading these molecules with chitinases (Punja and Zhang, 1993; Zhu et al., 2021). To counteract, fungal pathogens secrete chitin-binding proteins to protect their hyphae from hydrolysis and avoid activating escalated defence responses (Cunnac et al., 2009). Cas9-mediated knockout mutants of a *L. maculans* gene *(LmCBP1)* encoding chitin-binding protein resulting in two mutants with reduced pathogenicity in *B. napus* (Liu et al., 2020). Similarly, the RNP complex has been used to confirm the roles of glucosinolate biosynthetic genes of *B. oleracea*, like *ST5b-Bol026202* and *MYB34-Bol017062* in resistance to *Mycosphaerella brassicicola* (Abuyusuf et al., 2018) and modify effector genes (*OBR08294 and OBR06881*) in *Colletotrichum higginsianum*, a hemibiotrophic fungal pathogen responsible for anthracnose disease in *Brassicas*, to identify the potential effectors involved in the pathogenicity of the disease (Bhadauria et al., 2024).

Towards improving fungal resistance in *Brassica* crops, plant host factors, such as Calmodulin-binding transcription activators (CAMTAs), BnWRKY transcription factors in *B. napus* and PRRs also play important roles in various plant biological processes including biotic stress tolerance and disease resistance (Finkler et al., 2007; Jose et al., 2020; Noman et al., 2021; Chen et al., 2021). Several *CAMTA* genes have been identified in *B. napus*, from which *BnCAMTA3*, also found in *B. oleracea*, may play a significant role in resistance against *Sclerotinia* stem rot infection (Rahman et al., 2016). This assumption is based on observations on *S. sclerotiorum* resistance of *Arabidopsis* plants with the *CAMTA3* mutation (*Atcamta1-6*), where the *camta3* plants were more resistant to *S. sclerotiorum* compared to wild-type and the other *camta* mutant plants (Rahman et al., 2016).

Based on the understanding of plant defense mechanisms and available Brassica genomic data, many candidate resistance genes have been identified. CRISPR/Cas9 have been used to introduce mutations to these genes to confirm their functions. In some cases, modifying genes that negatively control host resistance result in mutants with improved resistance to fungal pathogens. The *WRKY* family is one of the largest plant transcription factor gene families, of which several members are involved in regulating the defence response (Chen et al., 2021). In *B. napus*, *BnWRKY28* and *BnWRKY33* have antagonistic roles in the response of *B. napus* to *S. sclerotiorum* invasion (Zhang et al., 2022), in which *BnWRKY28* on chromosome A03 *(BnaA03.WRKY28)* functions negatively in resistance to *S. sclerotiorum* in *B. napus*. The CRISPR/Cas9 system was used to mutate six sites in the target gene, resulting in the *BnaA03.WRKY28* knockout mutant lines with enhanced resistance to *S. sclerotiorum*, the organism responsible for stem canker (Zhang et al., 2022). By similar approach, improved resistance to *Sclerotinia* was also reported in transgenic *B. napus* plants containing *BnaWRKY70* mutants (Sun et al., 2018).

Plant host factors have different effects on fungal infection. During fungal infection, a lysin motif-containing PRR, chitin elicitor receptor kinase 1 (*CERK1*), perceives and binds to chito-oligosaccharides (COs) elicitors, which activates the downstream signalling cascade and, hence, mounts immune responses (Liu et al., 2012; Yang et al., 2022).While most RLKs have been reported to have positive roles in empowering plant resistance during fungal infection (Soltabayeva et al., 2022), a few RLKs were reported with adverse effects. A receptor-like protein kinase 902 (RLK-902) in *B. napus* has been suggested to assist *S. sclerotiorum* and *Botrytis cinerea* pathogenicity (Zhao et al., 2024). The function of RLK-902 in disease progression was confirmed in CRISPR/Cas9-based *RLK-902* knockout mutants in *B. napus* through pathogenicity tests with *S. sclerotiorum* and *B. cinerea.* The *RLK902* knockout lines significantly improved disease resistance without compromising plant growth and development (Zhao et al., 2024; Ye et al., 2024). Overexpression of lysine motif RLK 4 in *Sinapis alba*, an inactive kinase from *B. juncea*, significantly induced resistance to *Alternaria brassicicola* compared with susceptible *B. juncea* in the same conditions (De et al., 2021).

Incorporating CRISPR-Cas9 technology into *Brassica* breeding programs offers tremendous possibilities for creating resilient crop varieties. In contrast to traditional breeding techniques, which can be time-consuming and less accurate, CRISPR gene editing enables specific genetic alterations conferring the expected resistance in T0 generationand significantly reduce the time for developing germplasm of resistant cultivars.

### 2.2 Bacteria/protists

Diseases induced by bacteria and protists are among the many challenges facing the sustainable cultivation of *Brassica* crops globally. Although there are fewer examples of bacterial and protist diseases of *Brassica* crops than those caused by fungi, they still require careful management to prevent significant yield losses. Clubroot, caused by the biotrophic obligate parasite protist *Plasmodiophora brassicae* (Javed et al., 2023), is a widespread disease of canola and vegetable *Brassicas*, which is particularly devastating to the Canadian canola industry (Zheng et al., 2020b) with yield loss in canola ranging from 60% to 90% (Pageau et al., 2006). It has emerged as one of the biggest threats to canola production, and its management alone has forced significant resource investment (Botero-Ramirez et al., 2022). The bacterial pathogen *Xanthomonas campetris* pv. *campestris* (*Xcc*) causes black rot, a major disease primarily affecting vegetable *Brassicas*, such as *B. oleracea* var *capitata* (Vicente and Holub, 2013; Sun et al., 2021). In addition, several species of bacteria belonging to the genera *Erwinia, Pseudomonas* and *Pectobacterium* result in bacterial leaf spot and soft rot in various *Brassica* species (Ren et al., 2001; Takikawa and Takahashi, 2014; Klair et al., 2021). Coordinating with recent advance in genomics, CRISPR/Cas tools have accelerated the identification, validation and utilization of clubroot and black rot resistance genes.

Deploying genetically resistant cultivars has been one of the primary management strategies to control clubroot (Hwang et al., 2014). This is because there is currently no way to eradicate the pathogen from infested soil, and soil amendments only provide limited disease control (Hasan et al., 2021). Therefore, there have been significant efforts to identify sources of natural resistance and the genes underlying clubroot resistance (see Hasan et al., 2021 for a comprehensive review of clubroot resistance genes). used a CRISPR/Cas9-based vector system to introduce the clubroot *R* gene *Rcr1* into the susceptible *B. napus* line DH12075. The modified plants in T_2_ generation were selection marker-free and showed stable resistance to clubroot. Beside Rcr1, two resistant genes from *B. rapa*, *CRa* and *Crr1*, encoding Toll-Interleukin1 receptor/nucleotide-binding site/leucine-rich-repeat (TIR-NBS-LRR; TNL) have been isolated (Yu et al., 2016). By transcriptomic analysis and comparative analysis of cell wall components in clubroot resistant *B.napus*, and Tu et al. (2024) suggested that *Rcr1* and *Crr1^rutb^* in canola mediated the induction of lignin accumulations and possibly interact with genes involved in the phenylpropanoid pathway. Over 10 resistance genes and over 20 QTLs were identified within Brassica species, especially in *B. rapa* (Yu et al., 2016). Hu’s study (Hu et al, 2024) demonstrates an efficient Cas9-based platform to quickly introduce a clubroot resistant line by integrating the resistance gene into a susceptible plant and a Cas9-assisted breeding framework that help avoiding rounds of back crossing, with the resistant line achievable in just two generations.

Another recent study examining clubroot resistance identified a microRNA-target pair that was thought to regulate clubroot resistance in the *B. rapa* cultivar ECD04 (Zhou et al., 2024). The authors generated a CRISPR/Cas9 construct by cloning their sgRNA into the pYLCRISPR/Cas9 expression vector and used it to disrupt *Bna-APS4*, a sulphate adenylyl-transferase gene that is targeted by miR395a. Loss-of-function mutants displayed increased resistance toward clubroot, implicating *Bna-APS4* as a negative regulator of clubroot resistance (Zhou et al., 2024). These studies demonstrate multiple applications of CRISPR/Cas9 to investigate resistance mechanisms toward clubroot in *Brassica* species. Their findings deepen the understanding of resistance mechanisms and can support the accelerated identification of novel clubroot receptors or genes that mediate clubroot resistance.

Cas9 from Streptococcus canis (ScCas9) was successfully employed to introduce broad-spectrum resistance against *Fusarium* wilt, black rot and clubroot in *B. oleracea* by knocking out the *BoBPM6* and *BoDMR6* genes (Zhang et al., 2025a). The *BPM6 gene*, *BTB/POZ (Broad complex*, *Tramtrack*, *Bric-a-brac/Pox virus and Zinc finger)-MATH 6* (*BPM6*), was differently expressed and induced by *Fusarium* wilt and black rot of *B. oleracea*. Meanwhile, *Downy Mildew Resistant 6* (*DMR6*) has been known as a conserved S gene (Zhang et al. 2025a; Thomazella et al., 2021). Subsequent inoculation experiments with homozygous mutants in T_1_ generation resulted in decreased disease indexes (DIs), as compared to DIs for wild-type plants, after being challenged with the three pathogens. The *bodmr6* mutant presented significant DI changes with black rot and clubroot infection (from 79.3 to 55.1, and from 90.7 to 57.6), while the *bobpm6* showed significant DI decrease with Fusarium wilt and clubroot (from 65.4 to 14.5, and from 53.8 to 20.9). These results suggest Cas9-based editing as a powerful in breeding disease resistance in *B. oleracea.*

Another study by the same authors employed CRISPR/Cas9 to optimise the modification of a potential host susceptibility gene, *BoSWEET15b* (*SWEET15b* gene in *B. oleracea*), in the cabbage cultivar ‘Ohgane’ (Kaur et al., 2023b). In plants, Sugars Will Eventually be Exported Transporter (SWEET) proteins have been known to involve in biological processes (e.g. regulation of pollen development, nectar secretion, seed development, phloem loading, and leaf senescence) and poteintial regulatory roles under biotic and abiotic stress (Song et al., 2022). The Cas9 ribonucleoprotein and sgRNA were transferred via a PEG-mediated delivery system, and a 39% insertion/deletion frequency was achieved in *BoSWEET15b* (Kaur et al., 2023b). The search for black rot resistance in cabbage is sped up supported by advancement in genomics (Ma et al., 2025). Therefore, the finding by Kaur et al (2023a) laid the foundation for employing CRISPR/Cas9 to both characterise potential black rot resistance genes and breeding black rot resistance in *B. oleracea*. Although in its infancy, applying CRISPR/Cas technology to enhance resistance to bacterial pathogens in *Brassica* crops is a promising tool to accelerate future resistance improvements.

### 2.3 Viruses

Viral diseases can cause up to 84% yield loss in rapeseed (*B. napus* and *B. juncea*) (Jones, 2021) and 65% in vegetable *Brassicas* (Das et al, 2024), which are often caused by turnip mosaic virus (TuMV, family *Potyviridae*), cucumber mosaic virus (CMV; family *Bromoviridae*), cauliflower mosaic virus (CaMV, family *Caulimoviridae)*, Turnip yellow virus (TuYV), and Brassica yellow virus (BrYV) (Bruckner et al., 2024; Wu et al., 2024). The last two viruses belong to the genus *Polerovirus* of the family *Luteoviridae* (Kidanemariam and Abraham, 2023). In natural conditions, aphids are the most common vector for transmission of the mentioned viruses (Ng and Perry, 2004; Latham et al., 2003), and mixed infections with TuMV, TuYV and CaMV often occur in *Brassica* crops worldwide (Raybould et al., 1999; Broadbent, 2015; Latham et al., 2003; Z Meena et al., 2021; Bruckner et al., 2024).

While persistently transmitted viruses, such as luteoviruses and poleroviruses, or semi-persistently transmitted viruses, such as TuYV and CaMV, can be managed using chemical treatment (Fereres and Raccah, 2015). Such treatment is ineffective for non-persistent viruses, such as TuMV and CMV (Hooks and Fereres, 2006). Additionally, the widespread of TuMV in Brassica growing areas worldwide, especially in cultivated *B. napus* and *B. rapa*, has necessitated the search for TuMV resistance genes and breeding TuMV resistance (Walsh et al., 2023; Palukaitis and Kim, 2021). Virus infection induces layers of plant response, such as PTI, RNA silencing and PAMP-triggered response. Upon TuMV infection, Brassica species response and develop symptom differently depending on viral strains. Most of the naturally identified virus resistance is conferred by R genes. Being transmitted by more than 80 aphid species have made TuMV become a concern for *Brassica* crops worldwide (Nellist et al., 2022).

As a model for RNA virus study and its economic importance to canola and cabbage crops, many research groups have focus on TuMV genome structure and TuMV resistance. The intensive studies revealed 16 dominant resistant loci or quantitative trait loci (QTLs) associated with TuMV resistance in the A genome of *B. rapa*, 5 resistance loci in the A or C genome of *B. napus*, and one in *B. juncea* (Palukaitis and Kim, 2021). Among the mapped resistance loci, the dominant and recessive resistance genes, *ConTR01* and *retr01* shared the same loci with the eukaryotic initiation factor 4E (eIF4E) and eIF(Iso)4E, respectively (Rusholme et al., 2007). This effort has been facilitated by advancements in sequencing and gene editing technologies, and Brassica genomic data, which deepens the understanding of molecular interactions between virus and plant host factors during the viral infection. In TuMV, viral genome-linked protein (VPg) functions like 5’-cap binding to eukaryotic translation initiation factors (eIF) and initiates viral protein translation (Okade et al., 2009; Leoonard et al., 2000);. Mutations in the binding sites of viral VPg or plant host eIF affect virus infectivity (Leoonard et al., 2000; Zhang R. et al., 2022; Lee et al., 2023b). Resistance to potyviruses, including TuMV, conferred by natural and artificial mutated *eIF4E* and *eIF(iso)4E* in different plant species, including *A. thaliana* and *B. rapa*, was thoroughly reviewed by Sanfaçon (2015). Therefore, currently, there are two approaches to utilise CRISPR/Cas tool for introducing virus resistance trait in plant host, either modifying plant genome to interfere virus multiplication and movement or directly cleaving genome of the infecting virus. The Cas9 systems are often selected for the first approach, while Cas13 systems are used for the later approach (Nie et al., 2024; Ton, 2024; Robertson et al., 2022; Mahas et al., 2019).

With climate change happening, general virus control measures become less effective, of which the most reliable ones are host resistance genes and control of insect vectors (Jose et al., 2020). Additionally, RNA viruses are prone to mutations that lead to the rise of novel strains (McDonald and Lindie, 2002). New TuMV strains overcoming known resistances in B. napus was reported in Australia and Korea (Song et al., 2022; Guerret et al., 2017). These facts necessitate the CRISPR/Cas tool deployment in the arms race between viruses and *Brassica* crops.

Induced sequence-specific point mutations at *eIF(iso)4E* locus in *A. thaliana* showed complete resistance to the GFP-tagged infectious TuMV clone pCB-TuMV-GFP with no TuMV detection by viral GFP imaging and quantitative RT-PCR. At the same time, TuMV-GFP systemic infection was visible in wild-type *Arabidopsis* plants (Pyott et al., 2016). There was no difference in plant growth between the mutants and the wild-type in this study, implying no compensations for growth in the *eIF(iso)4E* mutant line. Following this approach, Lee et al (2023b) utilised CRISPR/Cas9 vector targeting the *eIF(iso)4E* genes (*Bra*035531, *Bra*039484, and *Bra*035393) of *B. rapa*. The eIF(iso)4E-T1 edited plants were successfully generated and exhibited resistance to TuMV under the experimental conditions. Both studies showed no phenotypic differences between the mutant lines and the wild-type plants of *Arabidopsis* and *B. rapa*. However, single-gene resistance is not durable and overcome easily by novel virus strains (Palukaitis and Kim, 2021). Fortunately, the advancement of Brassica genomic data has supported the search for new candidate resistance genes. Shopan et al (2020) revealed that new eIF subgroups, eIF2β, eIF2α, eIF2Bβ, eEF1A, and poly(A)-binding proteins (PABPs) could be the targets for antiviral strategies in *B. juncea*. Meanwhile, defect/mutated eIF2Bβ, eIF4E, eIF(iso)4E, eIF4G, and eIF(iso)4G have been found to confer recessive resistance to plant viral infections (Shopan et al., 2017, Shopan et al., 2020).

In addition to modifying the plant genome, virus resistance can be achieved by using RNA-targeting CRISPR/Cas systems to target and cleave viral genomes in infected plant hosts. A reprogrammed Cas9 protein from *Francisella novicida* (FnCas9) inhibited CMV in *Arabidopsis*, conferring virus resistance (Zhang et al., 2018a). The functionality of CRISPR-Cas13 systems in plant cells encouraged the use of this tool in tackling RNA plant viruses. Aman et al. (2018a) investigated the ability of the Cas13a effector from *Leptotrichia shahii* (LshCas13a) to target TuMV *in vitro* and *planta* on tobacco plants (*N. benthamiana)*. This study used a fusion clone of TuMV-GFP to visualise the virus movement in the plants expressing crRNA-LshCas13a. Four crRNAs targeting TuMV-GFP at the sites encoding for HC-pro, CP, GFP1 and GFP2 were cloned as uniplexes or a multiplex of 3 crRNAs (HC-pro, GFP1 and GFP2) into tobacco rattle virus (TRV) vectors. Transient expression assays with *Nicotiana benthamiana* leaves agro-infiltrated with a mixture of *A. tumacien* strain GV3010 containing TuMV-GFP, LshCas13a vector, and TRV-crRNAs showed an apparent reduction in GFP intensity at seven days post-inoculation compared with the control treatments with neither LshCas13a expression nor the TRV-crRNAs. In pCas13a-overexpressed tobacco plants, leaf agro-infiltration with TuMV-GFP and uniplex TRV-crRNAs or multiplex TRV-crRNA produced a similar result as the transient assay. Transgenic *A. thaliana* lines expressing crRNA- LshCas13a targeting HC-Pro also had reduced TuMV accumulation upon being infected with TuMV-GFP (Aman et al., 2018b). The results on both model plants showed higher TuMV inhibition efficiencies with crRNAs binding HC-pro and GFP-T2 targets compared to CP and GFP-T1 targets. The LshCas13a systems have been successful in inhibiting the tobacco mosaic virus (TMV), southern rice black-streaked dwarf virus (SRBSDV), and rice stripe mosaic virus (RSMV) invasion in *N. benthamiana* and rice (Zhang et al., 2019). Together, these studies have revealed the potential use of Cas13 tools to confer resistance to RNA viruses in monocot and dicot plants, advancing crop breeding strategies for RNA virus resistance.

## 3 Abiotic stress tolerance in *Brassica*

Climate change consequences have intensified abiotic stress on Brassica crops and vegetables. Stress conditions such as drought, high temperature, and high salinity adversely affect plan physiological, metabolic and biochemical processes, resulting in significant reduction in yield and productivity (Raza et al., 2021). Plants possess different mechanisms in response to each individual stress. Recent transcriptomic studies on Brassica under abiotic stress and application of CRISPR/Cas9 in elucidating the role of stress responsive genes involved have provided insights into the molecular mechanisms underlying the stress responses. The developing understanding on the key genes controlling these mechanisms will facilitate the application of modern molecular breeding techniques, such as genetically transformation and genome editing (Sato et al., 2024).

### 3.1 Drought tolerance

Drought is caused by several reasons, including low soil moisture, salinity, high and low temperature (Salehi-Lisar and Bakhshayeshan-Agdam, 2016). Drought stress worsened by climate change significantly challenges these crops, affecting growth, development, and productivity (Wang et al., 2024). Annually, drought causes a minimum 30% reduction in canola yield (Farooq et al., 2009). Therefore, it is crucial to study *Brassica* crops’ responses to drought stress and strategies to enhance their drought resistance.

Drought inhibits photosynthesis, reducing biomass and yield, and disrupts biochemical pathways (Liu et al., 2004; Wahab et al., 2022; Zahra et al., 2023). However, plants have evolved adaptations to combat drought stress effectively (Yang et al., 2021), primarily through regulating abscisic acid (ABA) biosynthesis and signalling cascades (Wei et al., 2025; Soma et al., 2021) Drought condition induces the expression of many ABA biosynthesis genes along with binding factors and transcription factors (TF), leading to an increase in ABA levels and ABA-orchestrating mechanisms (Muhammad Aslam et al., 2022). Regulating stomatal closure to avoid dehydration is the primary plant response to drought (Sato et al., 2024). However, long-term photosynthesis disruption due to stomatal closure increases oxidative stress, activating stress-responsive genes like superoxide dismutase, catalase, dehydrins, late embryogenesis abundant (LEA) and DELLA proteins (Sun et al., 2021; Wang et al., 2020). Therefore, the activation of reactive oxygen species (ROS) scavenging pathways and increased biosynthesis of the protective genes represent drought tolerance. Genes responsive to drought and abscisic acid (ABA) are vital for plant protection, influencing LEA proteins, chaperones, osmo-protectants, sugar and proline transporters, aquaporins, and ROS-detoxifying enzymes (Muhammad Aslam et al., 2022; Kim et al., 2024).

Tolerance to drought stress is a complex quantitative trait (Rahman et al., 2022). Generating knock-out mutants of key stress responsive genes by CRISPR/Cas9 is a time efficient approach to elucidate functions of genes responsive to drought stress, which have been demonstrated through multiple studies in Arabidopsis. ABA mediated stomatal closure is the popular target for improving drought tolerance in crops (Hyun, 2020). *OPEN STOMATA 2* (*OST2)* gene, which encodes a plasma membrane H^+^ATPase AHA1, activates many secondary transporters involve in ion and metabolite uptake and prevents ABA-mediated stomatal closure (Merlot et al., 2007). Osakabe et al (2016) successfully generated homozygous OST2 mutated Arabidopsis using CRISPR/Cas9. The homozygous line with 1-bp frameshift OST2 mutation showed a lower rate of transpirational water loss compared with that of the wild-type. Drought tolerance enhancement can be achieved by editing activation of the vacuolar H^+^-pyrophosphate (*AVP1*) regulatory gene, abscisic acid-responsive element binding (*AREB1*) gene, and silencing of the trehalase 1 (*TRE1*) gene, as well as editing of the *STL1* structural gene (Park et al., 2017a; Roca Paixão et al., 2019; Nuñez-Muñoz et al., 2021). Increased drought tolerance phenotypes include plant survival, growth, and development after drought treatment. In case of overexpressing *AVP1* gene, the modified plants survived and restored growth after 8 days without watering, while the wild type died after seven days (Park et al., 2017a). Regarding plant growth, the edited plants resulted in 2–5 fold increases in expression, additional four leaves, double size in single-leaf area, and enhanced drought tolerance compared to the wild-type (Park et al., 2017a). These preliminary studies on the model plants provide platforms for employing CRISPR/Cas9-assisted breeding towards drought tolerance in cultivated Brassica species.

The CRISPR-Cas9 systems are powerful tools to characterise functions of members of gene family contributing to stress response in an allotetraploid species, such as *B. napus*. As an example, CRISPR/Cas9 was deployed to edit *BnaA6.RGA. B. napus* contains 10 DELLA genes, including four homologs of RGA: *BnaA6.RGA*, *BnaC7.RGA*, *BnaA9.RGA*, and *BnaC9.RGA*. Previously, CRISPR/Cas9 technology was used to create mutants of these *BnaRGAs* (Yang et al., 2017). Subsequently, Wu et al. (2020) confirmed gain-of-function mutants of *BnaA6.RGA* and *BnaC7.RGA* among the *BnaRGA* mutants, which positively regulate drought tolerance in *B. napus*.

In a study, Lee et al (2023b) used CRISPR/Cas9 technology to investigate the role of the *ELONGATED HYPOCOTYL 5* (*HY5*) gene in *B. rapa* under endoplasmic reticulum (ER) stress conditions which generally result from abiotic stresses (eg., increasing temperature, drought, salinity and pathogen infection). Researchers targeted the *HY5* gene with sgRNAs and confirmed mutations. When subjected to ER stress using tunicamycin (TM), wild-type and *hy5* mutant plants showed increased growth inhibition with higher TM concentrations, but the *hy5* mutants had less severe inhibition. Staining methods revealed that *hy5* mutants produced lower reactive oxygen species levels under ER stress. Additionally, these mutants exhibited lower expression levels of genes related to the unfolded protein response and cell death. The study concludes that editing the *HY5* gene can reduce stress-related growth inhibition, potentially improving crop quality and yield.

In a recent study (Wang et al., 2024), using CRISPR/Cas9 to introduce a nonsynonymous substitution (M63I) in the target gene, researchers confirmed a transcription factor called *BnaA9.NF-YA7* (nuclear factor-Y) in *B. napus* that negatively affects drought tolerance. They used a genome-wide association study to pinpoint this factor, highlighting two specific SNPs within a CCAAT cis-element that reduced expression of the *B. napus* nuclear factor *YA7* (*BnaA9.NF-YA7*) under drought conditions. Additionally, they discovered a genetic substitution (M63I) that activates *BnaA4.DOR* inhibits abscisic acid (ABA)-induced stomatal closure, thereby affecting water regulation in the plant. Furthermore, the study revealed that *Bna.ABF3/4s* directly control the expression of *BnaA9.NF-YA7*. Interestingly, *BnaA9.NF-YA7* indirectly suppresses *Bna.ABF3/4s* expression through its regulation of *Bna.ASHH4s*. These findings underscore the role of *BnaA9.NF-YA7* is used to maintain ABA signal balance during drought stress in canola. The study suggests that targeting *BnaA9.NF-YA7* could be a promising strategy for breeding drought-tolerant varieties of *B. napus*.

Although some drought responsive genes have been cloned and functionally characterised in *B. napus* and *B. rapa*, the molecular signalling during stress response in Brassica species is largely unexplored (Wang et al., 2024). The dependence on Agrobacterium-mediated transformation of CRISPR/Cas delivery and genotype-dependence of transformant regeneration conditions may hindered the Cas-based breeding process. Moreover, exploring functions and roles of homologous genes controlling a complex trait like drought tolerance has recently initiated in Brassica along with the evolution of sequencing technologies (Zhang et al., 2023; Kayum et al., 2015). To date, CRIPSPR/Cas9 have been mainly utilised to elucidate functions of potential genes involved in the drought response.

### 3.2 Salinity and metal toxicity tolerance

Salinity and metal toxicity are other persistent problems affecting *Brassica* crop production. Like other abiotic stresses, they affect key physiological processes, ultimately affecting yield outcomes in these crops (Dahlawi et al., 2023; Pavlović et al., 2019). Losses to salinity stress have been reported to reach up to 50% (Chakraborty et al., 2016), while toxic concentrations of heavy metals significantly reduce plant development, resulting in severe yield and quality reduction (Dahlawi et al., 2023).

Salinity stress is commonly due to high concentration of Na^+^ and Cl^−^ in the soil, resulting in hyperosmotic and hyperionic conditions (Ismail et al., 2014; Yang et al, 2018). Salt stress tolerance in *Brassica* spp is a complex trait that varies among species, of which allotetraploid species (*B. juncea*, *B. carinata*, and *B. napus)* are relatively more tolerant to salt stress as compared to their diploid parents, such as *B. campestris*, *B. nigra*, and *B. oleracea* (Shahzad et al., 2022). In *B. napus*, salt stress induced photosynthesis reduction, leaf gas exchange, and high ROS production (Shahzad et al., 2022; Wani et al., 2013). The comparison between halophyte and glycophytic plants (*quinoa* and *Atriplex* versus sugar beet and bean) highlight typical features of salt stress tolerance, such as high K^+^ retention in leaf mesophyll associated with higher vacuolar Na^+^ sequestration and less H^+^ pumping.

The deployment of *CRISPR*/*Cas* systems in studying salinity tolerance in *Brassica* is an emerging field, with pioneering works mainly relying on the knowledge gained from the related model species *A. thaliana*. For instance, knock-down of transcription factors (TFs) WRKY3, WRKY4, WRKY66 and AITR (ABA-induced transcription repressor) using CRISPR/Cas9 resulted in severe salt stress in *A. thaliana*, indicating the critical role of regulatory elements in mediating salt tolerance in plants (Chen et al., 2021; Li et al., 2021a; Zhang et al., 2023). WRKY proteins are key regulators in developmental processes, such as trichome initiation, embryp morphogenesis, senescence, and plant hormone-mediated signal transduction processes by GA, ABA, or SA (Kayum et al., 2015). Orthologs of these genes have been found in *Brassica* species and can be potentially targeted to improve salt tolerance in these crops (Zhang et al., 2023). These findings enhance the understanding of the complicated genetic control of salinity tolerance in crops extending beyond the known mechanisms, such as osmotic regulation and ion sequestration (Shah et al., 2018). Rewiring the regulatory network where these genetic elements participate can potentially create novel salt-tolerant phenotypes valuable for breeding (Hetti-Arachchilage et al., 2022).

CRISPR/Cas has also helped investigate the interaction of salt stress with other trace elements essential for plant growth, including boron, which has been reported to alleviate salt stress in crops (Qu et al., 2024). In *B. napus*, boron application upregulated the expression of *BnaA2.HKT1*, a gene involved in sodium (Na) unloading in plant cells (Hua et al., 2024). CRISPR/Cas9-knockout mutants of this gene were severely affected by salt stress despite the presence of elevated boron concentration. This suggests that boron is a positive regulator of Na unloading mechanisms, increasing the plant’s tolerance to salt stress. This finding informs the implementation of an optimal soil fertilisation strategy to enhance salinity tolerance in *B. napus*, translating CRISPR/Cas research into an actual field management practice.

Similarly, CRISPR/Cas systems have proven useful in identifying genes involved in metal toxicity tolerance in *Brassica* crops. Loss of function mutation in the gene *BnaNRAMP1* using *CRISPR/Cas9* resulted in low cadmium (Cd) accumulation in *B. napus* plants (Zhang et al., 2025b). Further analysis indicates this gene is crucial in detoxifying Cd by reducing its toxic levels within plant cells. Another gene that regulates Cd absorption is *BnCUP1* (Yao et al., 2022), which was previously implicated in chelating excess copper in plant cells. The disruption of this gene using CRISPR/Cas9 in *B. napus* reduced the accumulation of Cd in both roots and shoots without negatively affecting the agronomic characteristics of the genome-edited plants. From the field experiment, in comparison with observations in the wild-type, *BnCUP1*-edited lines accumulated less Cd with reduction by 52% in roots and by 77% in shoots and increased in the biomass (by 42%) and yield (by 47%). Furthermore, the other key elements, including iron, zinc, and manganese, were maintained in typical concentrations, suggesting that *BnCUP1* does not affect the absorption of these elements to maintain normal plant growth. The created *BnCUP1*-edited lines are important germplasm for breeding Cd safe edible and fodder oilseed rape.

While applying the CRISPR/Cas systems for studying salt and metal toxicity tolerance in *Brassica* crops is still a developing field, the abovementioned studies provide the groundwork for effectively implementing genome editing strategies to develop cultivars better adapting to these stresses. This will be further supported by the continuous development of high-quality genomic resources (Amas et al., 2023), which enables faster identification of candidate genes for various agronomic traits, including tolerance to various stresses.

### 3.3 Extreme temperature tolerance

Under current climate change and global warming scenarios, the impact of higher temperatures on crop production, particularly *Brassica* crops, is a critical concern. Climate model simulations indicate that by 2100, the Earth’s average temperature may rise by 1.1 to 5.4°C, and projections suggest a 50% increase in drought-affected areas (Battisti and Naylor, 2009; Herring, 2012).

The upper threshold warm temperature varies based on plant species. In general, an increase by 5–10 °C exceeding a plant’s optimal growth temperature triggers ROS burst and irreversible cellular oxidative damage, especially in photosynthesis systems I and II (Kan et al., 2023). Numerous studies have shown that heat stress, often coupled with drought, significantly affects crop production by impacting plant metabolism, reproduction, and physiology (Nadeem et al., 2019; Kourani et al., 2022). Studies have reported substantial crop yield reductions (Frenck et al., 2011; Hasanuzzaman et al., 2014). As cool-season crops, *Brassicas* can be extremely sensitive to high temperatures, significantly impacting their production (Yu et al., 2014; Ahmed et al., 2020). Hence, due to global population growth, expanding crop varieties that can withstand environmental stresses is crucial, using traditional breeding methods and advanced biotechnological tools such as CRISPR/Cas9.

The CRISPR/Cas9 technology has been applied extensively to enhance heat tolerance in various crops, however, its application in *Brassica* species for this trait remains relatively limited. Heat shock proteins (HSPs) act as molecular chaperones that aid cellular survival by transporting, folding, and degrading proteins under heat stress (Xu et al., 2011). Increased expression of HSP70 genes has been shown to provide more excellent resistance to abiotic stresses, including high temperatures (Wang et al., 2016; Zhao et al., 2019). Under heat stress condition, heat stress transcription factors (HSFs) bind to with *HSP* gene promoters and interact with heat shock factor binding proteins (*HSBP*) genes. HSF-mediated stress tolerance is negatively regulated by HSBPs, which affects the DNA-binding capacity activation activity of HSFs (Muthusamy et al., 2023). Although, the loss-of-function mutant of *BrHSBP1* in B. rapa was indistinguishable from the *BrHSBP1* overexpressed line in heat-stress responsive phenotypes, the Muthusamy et al. (2023) suggested the positive role of BrHSBP1 in drought-stress response through supporting raffinose biosynthesis, through which enhanced yield and stress tolerance can be achieved.

Heat stress significantly reduces seed production in *Brassica* species by altering the typical structure of floral organs. In a mutation known as sepal carpal modification (scm) observed in *B. rapa*, four of the five sepals merge to form a ring structure enclosing abnormal stamens and a pistil, ultimately leading to diminished seed yield. This mutation affects homologues of the *BrAP2* gene, which are orthologous to the *APETALA* (*AP2*) gene in *Arabidopsis*. Researchers used CRISPR/Cas9 technology to generate knockout plants of four *BnAP2* gene homologues in rapeseed, aiming to explore their roles in sepal and petal development (Zhang et al., 2018b). In another study, the expression of *BrRH22*, a chloroplast-targeted DEAD-box RNA helicase, in cabbage (*B. rapa*) was markedly increased by heat, drought, salt or cold stress (Nawaz et al., 2018). *BrRH22* has been known to contribute positively to seed germination and plant vigor under varied abiotic stress conditions (Nawaz et al., 2018). DEAD-box RNA helicases (RHs) are nucleus-encoded chloroplast-targeted RNA-binding proteins (RBPs) involve in regulating chloroplast gene expression.

In conclusion, efforts to enhance heat tolerance through advanced biotechnological tools like CRISPR/Cas9 show promise. Future research should focus on expanding these technologies to commercial *Brassica* crops, such as *B. napus*, to mitigate the adverse effects of heat stress on yield and ensure food security in a changing climate. Integrating traditional breeding methods with cutting-edge biotechnology remains pivotal in developing resilient *Brassica* varieties capable of withstanding anticipated climatic challenges.

## 4 CRISPR gene editing in modern plant breeding

CRISPR genome editing can be integrated into plant breeding to improve resistance to biotic and abiotic stress. This involves a thorough literature review of the transcriptomic and genomic data to identify targets involved in regulating plant’s mechanisms to enhance resistance to biotic and abiotic stress (Kumar et al., 2023). Advances in plant molecular biology techniques have helped decipher the principles underlying plant signalling and regulatory pathways involved in abiotic and biotic stress responsiveness (Aroca and García, 2023). The co-occurrence of biotic and abiotic stresses sometimes results in the activation of signalling and regulatory pathways that are either unrelated, additive, or conflicting to the stress (Li et al., 2019; Yang et al., 2019). Transcriptome analysis of the plants exposed to individual and combined stresses identifies genes and transcriptional factors involved in the stress response (Rizhsky et al., 2004). On the other hand, techniques such as RNA-seq, genome-wide association studies (GWAS), and quantitative trait loci QTL mapping help to determine the significantly upregulated and down-regulated genes in plants (Meng et al., 2020). QTL mapping has identified factors involved in resistance to *Sclerotinia* stem rot on the chromosome locus *SRC06* (Wu et al., 2013). CRISPR can be used to validate the function of the candidate genes on the locus in a short time and choose the right gene responsible for providing resistance.

The next step to validate gene function is to create a knock-out in resistant or stable transformants of the gene in susceptible plants. CRISPR/Cas genome editing offers great potential for targeted gene editing in plants (Zhang et al., 2018). The outcome of these studies is then utilized in plant breeding through traditional breeding approaches such as backcrossing and selfing to incorporate the mutated gene (Kumar et al., 2023). The plants with mutated genes are then tested in the fields under real growth conditions and challenged by the abiotic or biotic stress to assess their yield and performance. This is done to ensure that the overall yield and growth of the plant is not affected by the desired mutation and the trait has successfully been passed on to the next generation.

Genome-wide identification and characterisation studies have identified several transcription factors (TFs) involved in biotic and abiotic stress responsiveness in *Brassica*. Some of these studies have highlighted the function of TFs such as BRASSINAZOLE-RESISTANT (BZR) (Saha et al., 2016), NUCLEAR FACTOR (NF-Y) (Xu et al., 2014), bZIPs, MYB, NAC, WRKYs and EREBPs (Meraj et al., 2020) in biotic and abiotic stresses as well as several regulatory pathways. BZR is a positive regulator of the brassinosteroid (BR) signaling pathway in different plant families (Saha et al., 2016). Members of the auxin-responsive transcription factors (ARF) transcription factors play role in altering the expression of genes involved in auxin, abscisic acid, MeJA, salicylic acid and ethylene (Li et al., 2021). These integrative analyses have facilitated the identification of several key genes involved in both biotic and abiotic stresses. Which genes in mutated forms/altering expression level can enhance plant growth during stress conditions, then, CRISPR/cas9 can be used for gene editing.

The genome of *Brassica* is polyploid, complex, and redundant, making it a challenge for genome editing. As there are multiple copies of the same genes, they need to be eliminated at the same time for functional studies (Khan et al., 2021). Moreover, the chromosomal rearrangement and epigenetic modification in polyploid plants often produce transcriptional changes such as activation of transposable elements, duplication, and neo-functionalization of genes and variable expression (Wendel et al., 2018), making it difficult to link genotype to phenotype and characterize gene function (May et al., 2023). In polyploid crops such as *Brassica*, the exact number of homologues and homeologous genes and their function remains unclear due to gene redundancy (Schaart et al., 2021).

The availability of well-curated databases such as Brassica Database (Cheng et al., 2011) for genetics and genomics, BrassicaEDB (Cheng et al., 2011) for gene expression database, and BrassicaTED (Murukarthick et al., 2014) a public database for the utilisation of miniature transposable elements in *Brassica* species, have helped to narrow down targets for gene editing. EnsemblePlants and NCBI’s GEO (functional genomics dataset) have further improved gene search by providing access to gene models and synteny with related species, such as *A. thaliana* (Bolser et al., 2016; Clough et al., 2024). These genetics and functional genomic databases not only provide a valuable tool for designing gRNAs with high specificity and minimal off-target effects but also differentiate between homeologous gene copies, which is a common challenge in polyploid Brassica genomes.

**Figure 1:**
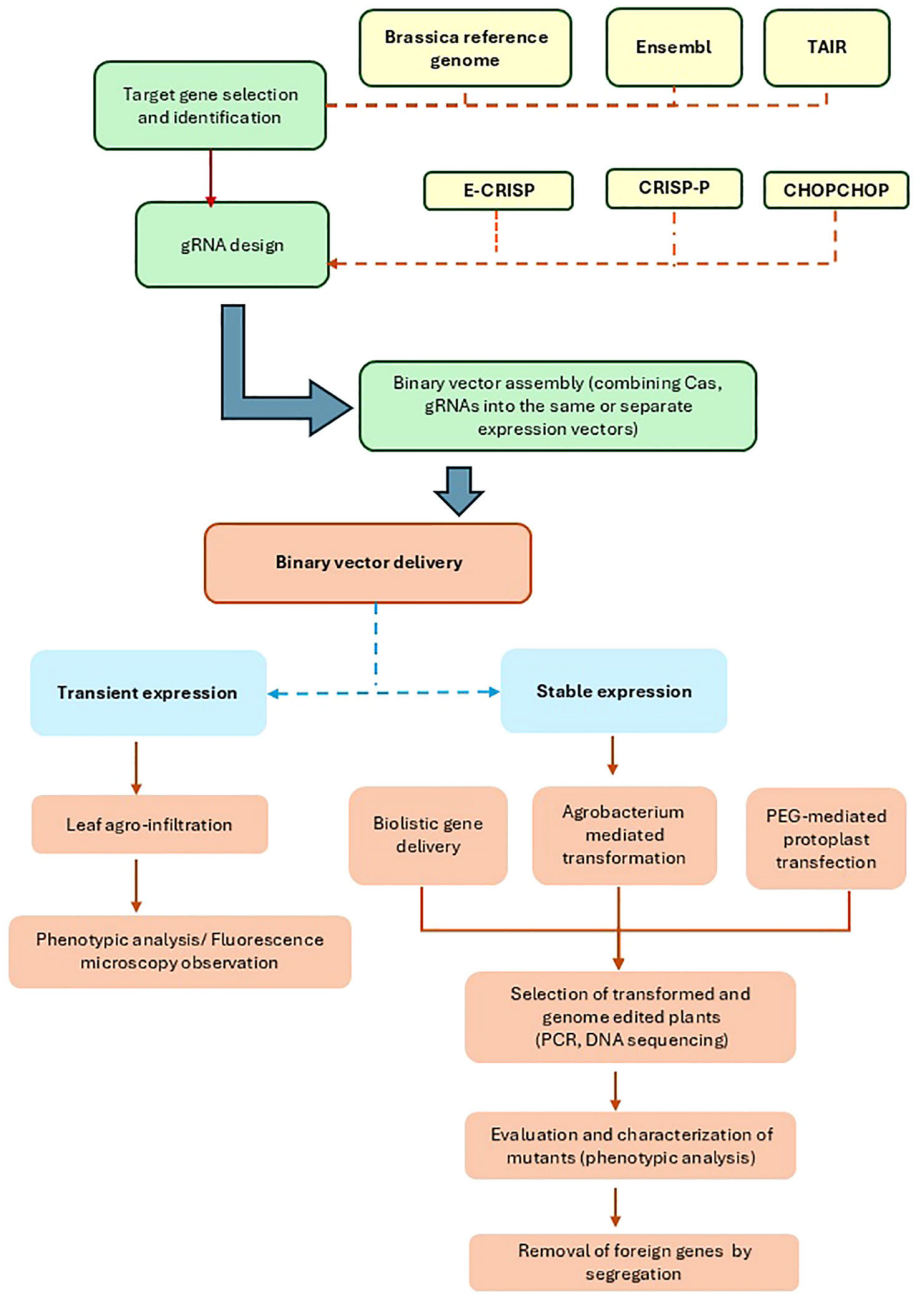
The flowchart shows a typical CRISPR/Cas-based genome editing pipeline in Brassica. Candidate genes are selected through transcriptomic and genomic analysis and used as targets for gRNA design. gRNA prediction tools, such as E-CRISP, CRISP-P and CHOPCHOP are used for designing gRNA. Then, CRISPR/Cas constructs specifically targeting the candidate genes are incorporated into transformation vectors. CRISPR/Cas constructs can be delivered and active through either transient (agro-infiltration) or stable transformation methods (biolistic gene delivery, Agrobacterium-mediated transformation and protoplast transformation). Following the transformation, modified plants are genotyped and selected on based on selection markers (e.g. fluorescence signal) and phenotypes. They are then multiplied and crossed for a few generations to confirm the transfer of the desired trait and removal of foreign DNA.

CRISPR-based tools have a significant advantage for genome editing in *Brassica* due to precise, homeolog-specific gene editing, providing researchers the ability to target genes and their copies on the subgenomes, evaluating their response to different stresses (Ahmad et al., 2023a). More advanced gene editing techniques, such as CRISPRa (CRISPR activation) of Resistance genes, and CRISPRi (CRISPR interference) of susceptible genes, can be effectively employed to improve disease resistance in *Brassica* crops (Zafar et al., 2020) **Figure 1**. Multiplex gene editing of JAGGED genes in *B. napus* through CRISPR has helped understand the role of several homeologs of the genes in pod shattering resistance. The results showed that a single mutation of one of the JAG genes on ChrA08 helped to improve pod shattering resistance (Zaman et al., 2019). In most reported studies, by targeting the key genes regulating the stress-responsive pathways, scientists can improve a plant’s ability to withstand adverse environmental conditions. Cas-edited mutants often have loss or gain functions, while some are fine-tune in their functions. The precision and flexibility in genome editing provide a huge potential for developing crops with enhanced yield, improved resistance to biotic and abiotic stress, and better quality.

## 5 Workflow for CRISPR/Cas application in *Brassica*

CRISPR/Cas-mediated genome editing in plants, including *Brassica* species, is often coupled with transformation. Therefore, the workflow for CRISPR/Cas-based modification in *Brassica* involves three main stages, as depicted in **Figure 1**: constructing a vector carrying an endonuclease system for targeted modifications, transforming vectors encoding the CRISPR/Cas components into plant cells, transgenic plant generating and evaluating the transformation and genome editing efficiency (Ahmad et al., 2023b). 

Firstly, developing a CRISPR/Cas construct may involve the following steps: identifying genomic targets, selecting a CRISPR/Cas tool, and designing and evaluating CRISPR/Cas vectors Hanna and Doench, 2020. The availability of Arabidopsis reference genome TAIR10 and the evolution of *Brassica* genomic data within recent years, especially an increasing number of published *Brassica* reference genomes and pangenomes, has benefited choices for the genetic improvement of *Brassica* crops via genome editing tools (Niu et al., 2024). Typical online Cas9-gRNA predicting tools are E-CRISP v.5.4 (Heigwer et al., 2014), CHOPCHOP (Labun et al., 2019), and Optimized CRISPR Plant Design Tool/CRISPR-P (http://cbi.hzau.edu.cn/cgi-bin/CRISPR; Liu et al., 2017; Lei et al., 2014). These tools employ published genome data as input for PAM scanning and gRNA designing. In 2022, Müller Paul et al., 2022 introduced CROPSR, a gRNA design and validation tool specialised for crop genomes, claiming this open-source tool written in Python 3.7 as the first developed tool for genome-wide generation and validation of sgRNA for crop genome editing and outperforming CHOPCHOP.

Following gRNA design, assembling CRISPR/Cas components is crucial in determining genome editing efficiency. A typical CRISPR/Cas expression vector can range from 9 to 16 kb (Zhang et al., 2024; Sun et al., 2018; Ma et al., 2015), containing Cas, gRNA, selection marker, and/or reporter gene sequences. Promoters control the expression of these genes. Choices of promoters, especially those controlling the expression of Cas enzymes and gRNAs, contribute to efficient genome editing. CaMV 35S, a strong constitutive promoter, is widely used for Cas expression in *Brassica* crops and vegetables (Villao-Uzho et al., 2023; Zhang D. et al., 2020). Use of other promoter types, such as tissue-specific promoters, inducible promoters, and Arabidopsis ubiquitin 10 promoter (Ubi10), were also reported (Tu et al., 2024; LeBlanc et al., 2018; Kurokawa et al., 2021; Papikian et al., 2019). Due to the small size of gRNA (20–30 nt), *A. thaliana* RNA polymerase III promoters (U3, U6) are often used to control the expression of gRNA cassettes in *Arabidopsis* and other *Brassica* species beside ubiquitin promoters (Li et al., 2022; Ma et al., 2019). In *Arabidopsis*, the gene editing efficiency of Cas9 driven by DD45 (subgroup of cysteine-rich peptide sequences, CRP) and the ribosomal protein S5A (RPS5A) promoters showed an increase in gene editing efficiency up to 30-fold compared to 35S and ubiquitin promoters (Ordon et al., 2020). Likewise, cell-specific promoters, such as pYAO and pEC, also showed similar effect (Chen et al., 2021, Chen et al., 2021; Wang et al., 2015). Cas and sgRNA(s) constructs of a CRISPR/Cas system can be expressed in the same or separate expression vectors. Other main components the expression vector includes are the localisation signal (NLS) and terminators of Cas and gRNA expression cassettes, which also attribute to efficient genome modification (Ordon et al., 2020).

For *Brassica* and other plant species, *Agrobacterium*-mediated transformation is the most commonly used method (Li et al., 2024; Li et al., 2022; Huang et al., 2020) for permanent and transient expression of CRISPR/Cas systems (Zhang K. et al., 2020; Aman et al., 2018a). Other methods such as PEG-mediated transformation and bombardment have been successfully applied for delivering CRISPR/Cas constructs directly into *Arabidopsis* protoplasts (Zhang Y. et al., 2022), *B. oleracea* (Romero and Pérez, 2024; Stelmach-Wityk et al., 2024; Stajič and Kunej, 2023), and *B. napus* (Li et al., 2021b). Yu et al. (2024) optimised a PEG-mediated protoplast transformation system for the transient expression of CRISPR/Cas9 vectors in *B. oleracea* L. (Chinese cabbage), Chinese kale and *B. rapa* (turnips) with improved protoplast yield and high transfection efficiencies (50 – 80%).

A non-transformation approach for CRISPR/Cas delivery, such as leaf infiltration and virus-based Cas-gRNA vectors, can be utilised to quickly evaluate the CRISPR/Cas system expression and skip many steps of *in vitro* plant regeneration. The small size of virus vectors (~ 1 kb) allows more efficient CRISPR/Cas delivery than Agrobacterium vectors (~ 8.9 kb). However, their package capacity is often below 4 kb (Varanda et al., 2021; Kirigia et al., 2014). Therefore, in a typical functional genomic study, gRNA constructs were cloned into virus-derived vectors, while Cas9 were delivered into plant cells via different vectors. Ali et al. (2015) developed the RNA2 genome of the bipartite genome of the tobacco rattle virus (TRV) into a vehicle for sgRNAs delivery in *Nicotiana benthamiana*. This TRV-mediated system also successfully delivered multiple sgRNAs into *Arabidopsis* leaves, leading to high mutation frequencies of targeted sequences (Ali et al., 2018). Other plant virus-derived CRISPR/Cas systems, also known as effective plant genome editing tools, such as pea early browning virus (PEBV), tobacco mosaic virus (TMV), and *Sonchus* yellow net rhabdovirus (SYNV), caused high frequency of target mutations in *N*. *benthamiana* (Ma, 2020; Ali et al., 2018). However, these have not been applied in *Brassica* plants.

**Figure 2:**
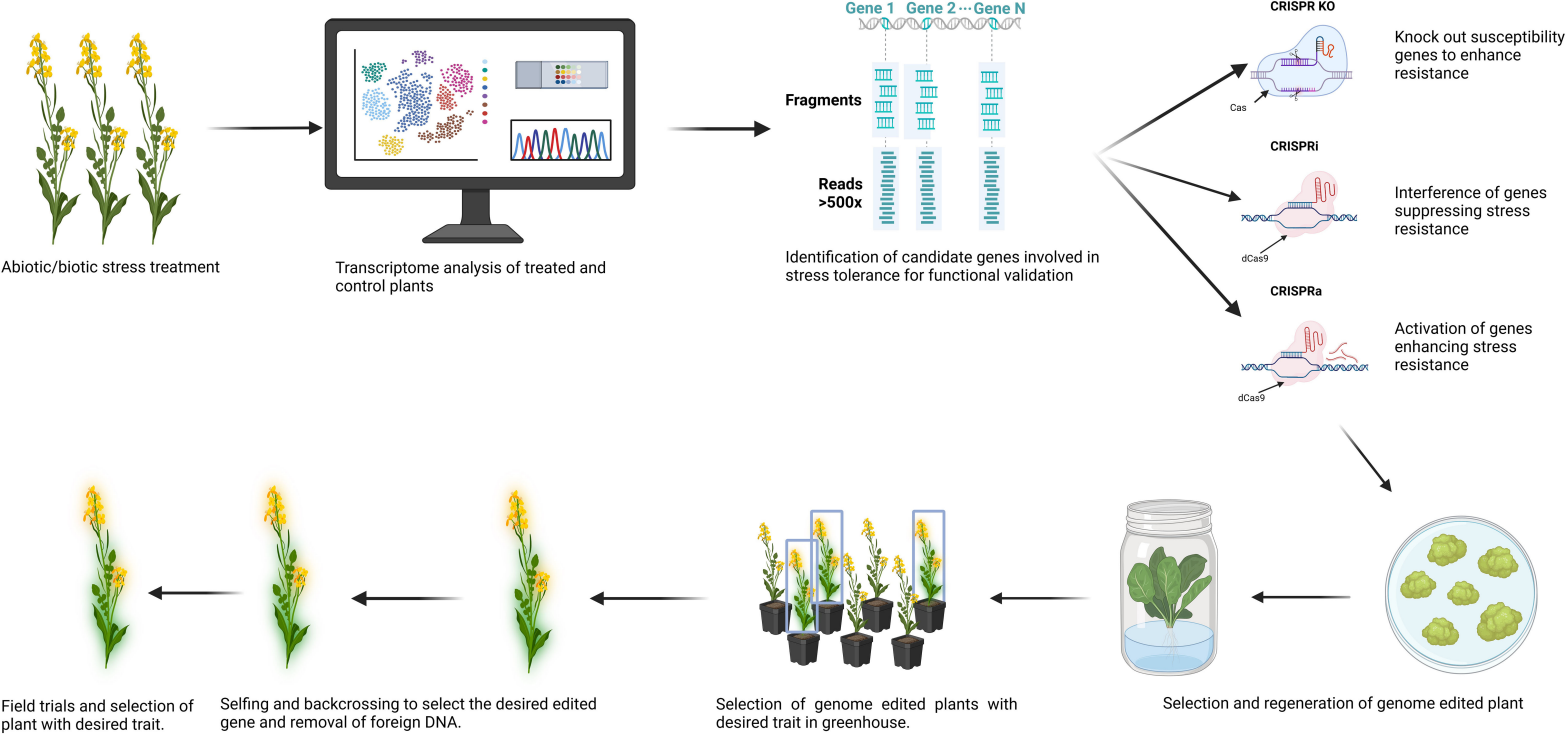
The workflow highlights key steps in employing advanced CRISPR/Cas tools (CIRSPR-KO, CRISPRi, and CRISPRa) in breeding abiotic/biotic stress tolerant Brassica plants, from genetic target identification, CRISPR/Cas vector construction and delivery, transformant selection and confirmation, characterization of genetic modifications, and the generation of “transgene-free” and homologous mutants of edited plants. Created in BioRender. Qayyum, Z. (2025) https://BioRender.com/3jb9miy.

To obtain a CRISPR/Cas mutant line, in most cases, transformed cells/tissues need to constitutively express CRISPR/Cas components, grow on selection media, and develop into whole plants (Romero and Pérez, 2024; Bao et al., 2019). The putative transformed plants are then genotyped and checked for the expression of the Cas and gRNA sequences using PCR and qPCR (Yu et al., 2024; Li et al., 2022; Hussain et al., 2018). Genome editing efficiency is calculated based on the number of plants containing targeted mutants and the total number of transgenic plants (Zhang et al., 2019). Mutated lines of the T_0_ population are then sequenced to confirm the CRISPR/Cas-induced mutations. For commercial, breeding or study purposes, several rounds of crossing and backcrossing of T_0_ progenies may be needed, as shown in **Figure 2**, to remove foreign genetic insertions in the plant genome, such as selection marker genes, gRNA and Cas sequences, and to obtain homozygous mutant plants as shown in **Figure 2** (Romero and Pérez, 2024). Transgene-free CRISPR/Cas-mutated plants can be directly obtained in T_0_ generation with protoplast-based genome editing by transient expression of CRISPR/Cas without incorporating the foreign gene construct into plant genome (Romero and Pérez, 2024). However, *Brassica* regeneration from protoplasts is more challenging than other explants, such as cotyledons and hypocotyl segments, and is a time-consuming process (Li et al., 2021b, Li et al, 2022). Therefore, *Agrobacterium*-mediated transformation using cotyledon or hypocotyl explants remains the dominant method (Zhang K. et al., 2020). Despite the availability of many regeneration protocols for the main *Brassica* crops (*B. napus*, *B. oleracea*, and *B. rapa*), their transformation efficiency and *in vitro* growth are mostly genotype-dependent (Farooq et al., 2019), presenting a major challenge for developing a CRISPR/Cas genome-edited *Brassica* crop.

## 6 Conclusion

CRISPR/Cas systems are efficient tools for exploring functions of *Brassica* genomes, especially the complex polyploid genomes of *B. napus*. These tools also help develop the knowledge of interactions between brassica hosts and their pathogens and the genetic mechanisms underlying abiotic stress responses in *Brassica* crops. This knowledge widens CRISPR/Cas-based approaches for the genetic control of *Brassica* phenotypes, improving resistance and resilience to biotic and abiotic stress. Although limited *Brassica* genes with known functions are being exploited for these breeding targets, several *Brassica* genomic resources are being constructed, and the evolution of *Brassica* multi-omics will provide more information for CRISPR/Cas genome editing interventions. Regarding tackling plant pathogens, CRISPR/Cas tools can target plant hosts or pathogens to improve plant defence capacity. At the same time, components of the CRISPR/Cas expression constructs are also being optimised for different research purposes and tailored for more efficient and controllable mutation inductions in *Brassica*. Genotype-dependence in transformation and regeneration of both *Brassica* explants and protoplasts remains a challenge, prolonging these processes. A transgene-free protoplast-based genome editing approach is still being pursued by many research groups to develop CRISPR/Cas-based edited Brassica crops for commercial release. Platforms for applying CRISPR/Cas genome editing tools in *Brassica* are urgently needed to accelerate the improvement of these crops under the negative consequences of climate change.

## References

[B1] Abuyusuf M. Robin A. H. K. Kim H.-T. Islam M. R. Park J.-I. Nou I.-S. (2018). Altered glucosinolate profiles and expression of glucosinolate biosynthesis genes in ringspot-resistant and susceptible cabbage lines. Int. J. Mol. Sci. 19.2833 doi: 10.3390/ijms19092833, PMID: 30235823 PMC6163659

[B2] Adwiyah R. Syaukat Y. Indrawan D. Mulyati H. (2023). Examining sustainable supply chain management (SSCM) performance in the palm oil industry with the triple bottom line approach. Sustainability 15, 13362. doi: 10.3390/su151813362

[B3] Ahmad N. Fatima S. Mehmood M. A. Zaman Q. U. Atif R. M. Zhou W.. (2023a). Targeted genome editing in polyploids: lessons from Brassica. Front. Plant Sci. 14,1152468. doi: 10.3389/fpls.2023.1152468, PMID: 37409308 PMC10318174

[B4] Ahmad N. Fatima S. Mehmood M. A. Zaman Q. U. Atif R. M. Zhou W.. (2023b). Targeted genome editing in polyploids: lessons from Brassica. Front. Plant Sci. 14, 1152468. doi: 10.3389/fpls.2023.1152468, PMID: 37409308 PMC10318174

[B5] Ahmed W. Xia Y. Li R. Bai G. Siddique K. H. M. Guo P. (2020). Non-coding RNAs: functional roles in the regulation of stress response in Brassica crops. Genomics 112, 1419–1424. doi: 10.1016/j.ygeno.2019.08.011, PMID: 31430515

[B6] Ali Z. Abul-Faraj A. Li L. Ghosh N. Piatek M. Mahjoub A.. (2015). Efficient virus-mediated genome editing in plants using the CRISPR/Cas9 system. Mol. Plant 8, 1288–1291. doi: 10.1016/j.molp.2015.02.011, PMID: 25749112

[B7] Ali Z. Eid A. Ali S. Mahfouz M. M. (2018). Pea early-browning virus-mediated genome editing via the CRISPR/Cas9 system in *Nicotiana benthamiana* and Arabidopsis. Virus Res. 244, 333–337. doi: 10.1016/j.virusres.2017.10.009, PMID: 29051052

[B8] Aman R. Ali Z. Butt H. Mahas A. Aljedaani F. Khan M. Z.. (2018a). RNA virus interference *via* CRISPR/Cas13a system in plants. Biol. 19. doi: 10.1186/s13059-017-1381-1, PMID: 29301551 PMC5755456

[B9] Aman R. Mahas A. Butt H. Aljedaani F. Mahfouz M. (2018b). Engineering RNA virus interference *via* the CRISPR/Cas13 machinery in Arabidopsis. Viruses 10. doi: 10.3390/v10120732, PMID: 30572690 PMC6315463

[B10] Amas J. C. Thomas W. J. W. Zhang Y. Edwards D. Batley J. (2023). Key advances in the new era of genomics-assisted disease resistance improvement of *Brassica* species. Phytopathology 113, 771–785. doi: 10.1094/PHYTO-08-22-0289-FI, PMID: 36324059

[B11] Aroca A. García I. (2023). Advances in plant molecular biology: Towards new challenges. J. Exp. Bot. 74, 5949–5954. doi: 10.1093/jxb/erad350, PMID: 37832938 PMC10575696

[B12] Asmamaw M. Zawdie B. (2021). Mechanism and applications of CRISPR/Cas-9-mediated genome editing. Biologics 15, 353–361. doi: 10.2147/BTT.S3264222, PMID: 34456559 PMC8388126

[B13] Bao A. Burritt D. J. Chen H. Zhou X. Cao D. Tran L.-S. P. (2019). The CRISPR/Cas9 system and its applications in crop genome editing. Crit. Rev. Biotechnol. 39, 321–336. doi: 10.1080/07388551.2018.1554621, PMID: 30646772

[B14] Basili M. Rossi M. A. (2018). *Brassica carinata*-derived biodiesel production: economics, sustainability and policies. The Italian case. J. Clean. Prod. 191, 40–47. doi: 10.1016/j.jclepro.2018.03.306

[B15] Battisti D. S. Naylor R. L. (2009). Historical warnings of future food insecurity with unprecedented seasonal heat. Science 323, 240–244. doi: 10.1126/science.1164363, PMID: 19131626

[B16] Bhadauria V. Han T. Li G. Ma W. Zhang M. Yang J.. (2024). A gln-tRNA-based CRISPR/Cas9 knockout system enables the functional characterization of genes in the genetically recalcitrant brassica anthracnose fungus *Colletotrichum higginsianum* . Int. J. Biol. Macromol. 254, 127953. doi: 10.1016/j.ijbiomac.2023.127953, PMID: 37951433

[B17] Bhattacharya S. (2019). Brassica-aphid interaction: Challenges and prospects of genetic engineering for integrated aphid management. Physiol. Mol. Plant Pathol. 108, 101442. doi: 10.1016/j.pmpp.2019.101442

[B18] Boivin K. Acarkan A. Mbulu R. S. Clarenz O. Schmidt R. (2004). The *Arabidopsis* genome sequence as a tool for genome analysis in *Brassicaceae.* A comparison of the *Arabidopsis* and *Capsella rubella* genomes. Plant Physiol. 135, 735–744. doi: 10.1104/pp.104.040030, PMID: 15208421 PMC514111

[B19] Bolser D. Staines D. M. Pritchard E. Kersey P. (2016). Ensembl plants: integrating tools for visualizing, mining, and analyzing plant genomics data. Plant bioinformatics: Methods Protoc., 115–140. doi: 10.1007/978-1-4939-3167-5_6, PMID: 26519403

[B20] Borges C. E. Von dos Santos Veloso R. da Conceição C. A. Mendes D. S. Ramirez-Cabral N. Y. Z. Shabani F.. (2023). Forecasting *Brassica napus* production under climate change with a mechanistic species distribution model. Sci. Rep. 13, 12656. doi: 10.1038/s41598-023-38910-3, PMID: 37542082 PMC10403512

[B21] Botero-Ramirez A. Hwang S. F. Strelkov S. E. (2022). Effect of clubroot (*Plasmodiophora brassicae*) on yield of canola (*Brassica napus*). Can. J. Plant Pathol. 44, 372–385. doi: 10.1080/07060661.2021.1989801

[B22] Broadbent L. (2015). *Investigation of virus diseases of Brassica crops* (No. 14) (Cambridge, UK: Cambridge University Press).

[B23] Bruckner F. P. Barbosa T. M. C. Eiras M. Zanardo L. G. Awasthi L. P. (2024). “Chapter 51 - broccoli, cabbage and cauliflower,” in Viral diseases of field and horticultural crops ed. Awasthi L. P. (London, UK: Academic Press), 427–436. doi: 10.1016/B978-0-323-90899-3.00022-7

[B24] Carroll D. (2008). Progress and prospects: Zinc-finger nucleases as gene therapy agents. Gene Ther. 15, 1463–1468. doi: 10.1038/gt.2008.145, PMID: 18784746 PMC2747807

[B25] Cathomen T. Keith J. J. (2008). Zinc-finger nucleases: the next generation emerges. Mol. Ther. 16, 1200–1207. doi: 10.1038/mt.2008.114, PMID: 18545224

[B26] Chakraborty K. Sairam R. K. Bhaduri D. (2016). Effects of different levels of soil salinity on yield attributes, accumulation of nitrogen, and micronutrients in *Brassica* spp. J. Plant Nutr. 39, 1026–1037. doi: 10.1080/01904167.2015.1109105

[B27] Charpentier E. Richter H. van der Oost J. White M. F. (2015). Biogenesis pathways of RNA guides in archaeal and bacterial CRISPR-Cas adaptive immunity. FEMS Microbiol. Rev. 39, 428–441. doi: 10.1093/femsre/fuv023, PMID: 25994611 PMC5965381

[B28] Chen H. Wang Y. Liu J. Zhao T. Yang C. Ding Q.. (2021). Identification of WRKY transcription factors responding to abiotic stresses in *Brassica napus* L. Planta 255, 3. doi: 10.1007/s00425-021-03733-x, PMID: 34837557

[B29] Chen K. Wang Y. Zhang R. Zhang H. Gao C. (2019). CRISPR/Cas genome editing and precision plant breeding in agriculture. Annu. Rev. Plant Biol. 70, 667–697. doi: 10.1146/annurev-arplant-050718-100049, PMID: 30835493

[B30] Chen S. Zhang N. Zhou G. Hussain S. Ahmed S. Tian H.. (2021). Knockout of the entire family of *AITR* genes in *Arabidopsis* leads to enhanced drought and salinity tolerance without fitness costs. BMC Plant Biol. 21, 1–15. doi: 10.1186/S12870-021-02907-9/FIGURES/9, PMID: 33726681 PMC7967987

[B31] Cheng F. Liu S. Wu J. Fang L. Sun S. Liu B.. (2011). BRAD, the genetics and genomics database for Brassica plants. BMC Plant Biol. 11, 1–6. doi: 10.1186/1471-2229-11-136, PMID: 21995777 PMC3213011

[B32] Chisholm S. T. Coaker G. Day B. Staskawicz B. J. (2006). Host-microbe interactions: shaping the evolution of the plant immune response. Cell 124, 803–814. doi: 10.1016/j.cell.2006.02.008, PMID: 16497589

[B33] Christian M. L. Demorest Z. L. Starker C. G. Osborn M. J. Nyquist M. D. Zhang Y.. (2012). Targeting G with TAL effectors: a comparison of activities of TALENs constructed with NN and NK repeat variable di-residues. PloS One 7, e45383. doi: 10.1371/journal.pone.0045383, PMID: 23028976 PMC3454392

[B34] Clough E. Barrett T. Wilhite S. E. Ledoux P. Evangelista C. Kim I. F.. (2024). NCBI GEO: archive for gene expression and epigenomics data sets: 23-year update. Nucleic Acids Res. 52, D138–D144. doi: 10.1093/nar/gkad965, PMID: 37933855 PMC10767856

[B35] Cox D. B. T. Gootenberg J. S. Abudayyeh O. O. Franklin B. Kellner M. J. Joung J.. (2017). RNA editing with CRISPR-cas13. Science 358, 1019–1027. doi: 10.1126/science.aaq0180, PMID: 29070703 PMC5793859

[B36] Cunnac S. Lindeberg M. Collmer A. (2009). *Pseudomonas syringae* type III secretion system effectors: repertoires in search of functions. Curr. Opin. Microbiol. 12, 53–60. doi: 10.1016/j.mib.2008.12.003, PMID: 19168384

[B37] Dahlawi S. Sadiq M. Sabir M. Farooqi Z. U. R. Saifullah Q. A.A. Faraj T. K. (2023). Differential response of *Brassica* cultivars to potentially toxic elements and their distribution in different plant parts irrigated with metal-contaminated water. Sustainability 15.1966 doi: 10.3390/SU15031966

[B38] Dalio R. J. D. Paschoal D. Arena G. D. Magalhães D. M. Oliveira T. S. Merfa M. V.. (2021). Hypersensitive response: From NLR pathogen recognition to cell death response. Ann. Appl. Biol. 178, 268–280. doi: 10.1111/aab.12657

[B39] Daniel K. A. M. Muindi E. M. D. Muti S. M. D. (2023). Cabbage (*Brassica oleracea*) production in Kenya: a review of its economic importance, ecological requirement and production constraints. Int. J. Plant Soil Sci. 35, 245–254. doi: 10.9734/ijpss/2023/v35i183287

[B40] Das S. Kundu A. Podder S. (2024). Impact of biotic stresses on the Brassicaceae family and opportunities for crop improvement by exploiting genotyping traits. Planta 259, 97. doi: 10.1007/s00425-024-04379-1, PMID: 38520529

[B41] De A. Maity A. Mazumder M. Mondal B. Mukherjee A. Ghosh S. (2021). Overexpression of *LYK4*, a lysin motif receptor with non-functional kinase domain, enhances tolerance to *Alternaria brassicicola* and increases trichome density in *Brassica juncea* . Plant Sci. 309, 110953. doi: 10.1016/j.plantsci.2021.110953, PMID: 34134846

[B42] FAOSTAT (2023). Crops and livestock products. Available online at: www.fao.org/faostat/en/data/QCL/visualize (Accessed November 2024).

[B43] Farooq N. Nawaz M. A. Mukhtar Z. Ali I. Hundleby P. Ahmad N. (2019). Investigating the *in vitro* regeneration potential of commercial cultivars of *Brassica* . Plants 8, 558. doi: 10.3390/plants8120558, PMID: 31795525 PMC6963692

[B44] Farooq M. Wahid A. Kobayashi N. Fujita D. Basra S. M. A. (2009). Plant drought stress: effects, mechanisms and management. Agron. Sustain. Dev. 29, 185–212. doi: 10.1051/agro:2008021

[B45] Fereres A. Raccah B. (2015). Plant virus transmission by insects. eLS.

[B46] Finkler A. Ashery-Padan R. Fromm H. (2007). CAMTAs: Calmodulin-binding transcription activators from plants to human. FEBS Lett. 581, 3893–3898. doi: 10.1016/j.febslet.2007.07.051, PMID: 17689537

[B47] Francisco M. Tortosa M. Martínez-Ballesta M. d. C. Velasco P. García-Viguera C.. (2017). Nutritional and phytochemical value of *Brassica* crops from the agri-food perspective. Ann. Appl. Biol. 170, 273–285. doi: 10.1111/aab.12318

[B48] Frenck G. van der Linden L. Mikkelsen T. N. Brix H. Jørgensen R. B. (2011). Increased [CO_2_] does not compensate for negative effects on yield caused by higher temperature and [O_3_] in *Brassica napus* L. Eur. J. Agron. 35, 127–134. doi: 10.1016/j.eja.2011.05.004

[B49] Greer S. F. Hackenberg D. Gegas V. Mitrousia G. Edwards D. Batley J.. (2021). Quantitative trait locus mapping of resistance to turnip yellows virus in *Brassica rapa* and *Brassica oleracea* and introgression of these resistances by resynthesis into allotetraploid plants for deployment in *Brassica napus* [Original Research. Front. Plant Sci. 12. doi: 10.3389/fpls.2021.781385, PMID: 34956278 PMC8703028

[B50] Guerret M. G. L. Nyalugwe E. P. Maina S. Barbetti M. J. van Leur J. A. G. Jones R. A. C. (2017). Biological and molecular properties of a Turnip mosaic virus (TuMV) strain that breaks TuMV resistances in *Brassica napus* . Plant Dis. 101, 674–683. doi: 10.1094/pdis-08-16-1129-re, PMID: 30678573

[B51] Guo Y. Zhao G. Gao X. Zhang L. Zhang Y. Cai X.. (2023). CRISPR/Cas9 gene editing technology: a precise and efficient tool for crop quality improvement. Planta 258, 36. doi: 10.1007/s00425-023-04187-z, PMID: 37395789

[B52] Hagit H. Steffen O. Anton R. Shany Ishgur G. Gur P. Julia K.. (2024). Rapid, direct, and sequence-specific identification of RNA viruses in various crop plants using CRISPR/Cas13a. bioRxiv 2024, 2002.2022.581525. doi: 10.1101/2024.02.22.581525

[B53] Hanna R. E. Doench J. G. (2020). Design and analysis of CRISPR–Cas experiments. Nat. Biotechnol. 38, 813–823. doi: 10.1038/s41587-020-0490-7, PMID: 32284587

[B54] Hasan J. Megha S. Rahman H. (2021). Clubroot in *Brassica*: Recent advances in genomics, breeding, and disease management. Genome 64, 735–760. doi: 10.1139/gen-2020-0089, PMID: 33651640

[B55] Hasanuzzaman M. Nahar K. Alam M. M. Fujita M. (2014). Modulation of antioxidant machinery and the methylglyoxal detoxification system in selenium-supplemented *Brassica napus* seedlings confers tolerance to high temperature stress. Biol. Trace Element Res. 161, 297–307. doi: 10.1007/s12011-014-0120-7, PMID: 25249068

[B56] Heigwer F. Kerr G. (2014). and Boutros, M. E-CRISP: fast CRISPR target site identification. Nat. Methods 11, 122–123. doi: 10.1038/nmeth.2812, PMID: 24481216

[B57] Hetti-Arachchilage M. Challa G. S. Marshall-Colón A. (2022). Rewiring network plasticity to improve crops. Plant Breed. Rev. 45, 143–183. doi: 10.1002/9781119828235.CH3

[B58] Herring D. (2012).Climate change: Global temperature projections. Available online at: www.climate.gov/news-features/understanding-climate/climate-change-global-temperature (Accessed November 15, 2024).

[B59] Hooks C. R. R. Fereres A. (2006). Protecting crops from non-persistently aphid-transmitted viruses: A review on the use of barrier plants as a management tool. Virus Res. 120, 1–16. doi: 10.1016/j.virusres.2006.02.006, PMID: 16780985

[B60] Hu H. Yu F. (2024). Studies on the temporal, structural, and interacting features of the clubroot resistance gene Rcr1 using CRISPR/Cas9-based systems. Hortic. Plant J. 10, 1035–1048. doi: 10.1016/j.hpj.2024.04.001

[B61] Hu H. Zhang Y. Yu F. (2024). A CRISPR/Cas9-based vector system enables the fast breeding of selection-marker-free canola with *Rcr1*-rendered clubroot resistance. J. Exp. Bot. 75, 1347–1363. doi: 10.1093/jxb/erad471, PMID: 37991105 PMC10901203

[B62] Hua Y. Pei M. Song H. Liu Y. Zhou T. Chao H.. (2024). Boron confers salt tolerance through facilitating *BnaA2.HKT1*-mediated root xylem Na+ unloading in rapeseed (*Brassica napus* L.). Plant J. 120, 1326–1342. doi: 10.1111/TPJ.17052, PMID: 39453388

[B63] Huang H. Cui T. Zhang L. Yang Q. Yang Y. Xie K.. (2020). Modifications of fatty acid profile through targeted mutation at *BnaFAD2* gene with CRISPR/Cas9-mediated gene editing in *Brassica napus* . Theor. Appl. Genet. 133, 2401–2411. doi: 10.1007/s00122-020-03607-y, PMID: 32448919

[B64] Hussain B. Lucas S. J. Budak H. (2018). CRISPR/Cas9 in plants: at play in the genome and at work for crop improvement. Brief. Funct. Genomics 17, 319–328. doi: 10.1093/bfgp/ely016, PMID: 29912293

[B65] Hwang S. F. Howard R. J. Strelkov S. E. Gossen B. D. Peng G. (2014). Management of clubroot (*Plasmodiophora brassicae*) on canola (*Brassica napus*) in western Canada. Can. J. Plant Pathol. 36, 49–65. doi: 10.1080/07060661.2013.863806

[B66] Hyun T. K. (2020). CRISPR/Cas-based genome editing to improve abiotic stress tolerance in plants. Bot. Serbica 44, 121–127. doi: 10.2298/BOTSERB2002121H

[B67] Iqbal Z. Iqbal M. S. Hashem A. Abd_Allah E. F. Ansari M. I. (2021). Plant defense responses to biotic stress and its interplay with fluctuating dark/light conditions. Front. Plant Sci. 12, 631810. doi: 10.3389/fpls.2021.631810, PMID: 33763093 PMC7982811

[B68] Ismail A. Takeda S. Nick P. (2014). Life and death under salt stress: Same players, different timing? J. Exp. Bot. 65, 2963–2979. doi: 10.1093/jxb/eru159, PMID: 24755280

[B69] Javed M. A. Schwelm A. Zamani-Noor N. Salih R. Silvestre Vañó M. Wu J.. (2023). The clubroot pathogen *Plasmodiophora brassicae*: A profile update. Mol. Plant Pathol. 24, 89–106. doi: 10.1111/mpp.13283, PMID: 36448235 PMC9831288

[B70] Jiang F. Doudna J. A. (2017). CRISPR–Cas9 structures and mechanisms. Annu. Rev. Biophys. 46, 505–529. doi: 10.1146/annurev-biophys-062215-010822, PMID: 28375731

[B71] Jinek M. Chylinski K. Fonfara I. Hauer M. Doudna J. A. Charpentier E. (2012). A programmable dual-RNA–guided DNA endonuclease in adaptive bacterial immunity. Science 337, 816–821. doi: 10.1126/science.1225829, PMID: 22745249 PMC6286148

[B72] Jones R. A. C. (2021). Global plant virus disease pandemics and epidemics. Plants 10, 233. doi: 10.3390/plants10020233, PMID: 33504044 PMC7911862

[B73] Jose J. Ghantasala S. Roy Choudhury S. (2020). *Arabidopsis* transmembrane receptor-like kinases (RLKs): A bridge between extracellular signal and intracellular regulatory machinery. Int. J. Mol. Sci. 21, 4000. doi: 10.3390/ijms21114000, PMID: 32503273 PMC7313013

[B74] Kan Y. Mu X. R. Gao J. Lin H. X. Lin Y. (2023). The molecular basis of heat stress responses in plants. Mol. Plant 16, 1612–1634. doi: 10.1016/j.molp.2023.09.013, PMID: 37740489

[B75] Kaur C. Lee M. Jeon Y. Lee G. J. (2023b). Enhancing black-rot resistance in cabbage using CRISPR/Cas9 system. Hortic. Sci. Technol. 41, 423.

[B76] Kaur C. Lee M. Yao J. Nam D. Lee G. J. (2023a). CRISPR/Cas12-mediated detection of *Xanthomonascampestris* pv. *campestris* (race 1 and 4) for improved pathogen diagnosis in cabbage. Hortic. Sci. Technol. 41, 338.

[B77] Kayum M. A. Jung H. J. Park J. I. Ahmed N. U. Saha G. Yang T. J.. (2015). Identification and expression analysis of WRKY family genes under biotic and abiotic stresses in Brassica rapa. Mol. Genet. Genom. 290, 79–95. doi: 10.1007/s00438-014-0898-1, PMID: 25149146

[B78] Khan M. H. U. Hu L. Zhu M. Zhai Y. Khan S. U. Ahmar S.. (2021). Targeted mutagenesis of EOD3 gene in Brassica napus L. regulates seed production. J. Cell. Physiol. 236, 1996–2007. doi: 10.1002/jcp.29986, PMID: 32841372

[B79] Kidanemariam D. Abraham A. (2023). “Chapter 3 luteoviruses,” in Plant RNA viruses, ed. Gaur R. K. Patil B. L. Selvarajan R.. (London, UK: Selvarajan (Academic Press), 57–77.

[B80] Kim J. S. Kidokoro S. Yamaguchi-Shinozaki K. Shinozaki K. (2024). Regulatory networks in plant responses to drought and cold stress. Plant Physiol. 195, 170–189. doi: 10.1093/plphys/kiae105, PMID: 38514098 PMC11060690

[B81] Kirigia D. Runo S. Alakonya A. (2014). A virus-induced gene silencing (VIGS) system for functional genomics in the parasitic plant *Striga hermonthica* . Plant Methods 10, 1–8. doi: 10.1186/1746-4811-10-16, PMID: 24932208 PMC4055913

[B82] Kirkegaard J. A. Lilley J. M. Berry P. M. Rondanini D. P. (2021). “Canola,” in Crop physiology case histories for major crops. Eds. Sadras V. O. Calderini D. F. (London, UK: Academic Press), 518–549.

[B83] Klair D. Boluk G. Silva J. Arizala D. Dobhal S. Arif M. (2021). First report of bacterial soft rot disease on pak choi (*Brassica rapa* subsp. *chinensis*) caused by *Pectobacterium brasiliense* in the United States. Plant Dis. 105. doi: 10.1094/PDIS-08-20-1854-PDN PMC8446085 33728952

[B84] Kourani M. Mohareb F. Rezwan F. I. Anastasiadi M. Hammond J. P. (2022). Genetic and physiological responses to heat stress in *Brassica napus* . Front. Plant Sci. 13, 832147. doi: 10.3389/fpls.2022.832147, PMID: 35449889 PMC9016328

[B85] Kumar M. Prusty M. R. Pandey M. K. Singh P. K. Bohra A. Guo B.. (2023). Application of CRISPR/Cas9-mediated gene editing for abiotic stress management in crop plants. Front. Plant Sci. 14, 1157678. doi: 10.3389/fpls.2023.1157678, PMID: 37143874 PMC10153630

[B86] Kurokawa S. Rhaman H. Yamanaka N. Ishizaki C. Islam S. Aiso T.. (2021). A simple heat treatment increases spCas9-mediated mutation efficiency in arabidopsis. Plant Cell Physiol. 62, 1676–1686. doi: 10.1093/pcp/pcab123, PMID: 34347875

[B87] Labun K. Montague T. G. Krause M. Torres Cleuren Y. N. Tjeldnes H. Valen E. (2019). CHOPCHOP v3: expanding the CRISPR web toolbox beyond genome editing. Nucleic Acids Res. 47, W171–W174. doi: 10.1093/nar/gkz365, PMID: 31106371 PMC6602426

[B88] Latham L. J. Smith L. J. Jones R. A. C. (2003). Incidence of three viruses in veget able brassica plantings and associated wild radish weeds in south-west Australia. Australas. Plant Pathol. 32, 387–391. doi: 10.1071/ap03031

[B89] Leoonard S. Plante D. Wittmann S. Daigneault N. Fortin M. G. Laliberte J. F. (2000). Complex formation between potyvirus VPg and translation eukaryotic initiation factor 4E correlates with virus infectivity. J. Virol. 74, 7730–7737. doi: 10.1128/jvi.74.17.7730-7737.2000, PMID: 10933678 PMC112301

[B90] LeBlanc C. Zhang F. Mendez J. Lozano Y. Chatpar K. Irish V. F.. (2018). Increased efficiency of targeted mutagenesis by CRISPR/Cas9 in plants using heat stress. Plant J. 93, 377–386. doi: 10.1111/tpj.13782, PMID: 29161464

[B91] Lee Y. R. Ko K. S. Lee H. E. Lee E. S. Han K. Yoo J. Y.. (2023b). CRISPR/Cas9-mediated *HY5* gene editing reduces growth inhibition in Chinese cabbage (*Brassica rapa*) under ER Stress. Int. J. Mol. Sci. 24, 13105. doi: 10.3390/ijms241713105, PMID: 37685921 PMC10487758

[B92] Lee Y. R. Siddique M. I. Kim D. S. Lee E. S. Han K. Kim S. G.. (2023a). CRISPR/Cas9-mediated gene editing to confer turnip mosaic virus (TuMV) resistance in Chinese cabbage (*Brassica rapa*). Hortic. Res. 10, uhad078. doi: 10.1093/hr/uhad078, PMID: 37323233 PMC10261878

[B93] Lei Y. Lu L. Liu H. Y. Li S. Xing F. Chen L. L. (2014). CRISPR-P: a web tool for synthetic single-guide RNA design of CRISPR-system in plants. Mol. Plant 7, 1494–1496. doi: 10.1093/mp/ssu044, PMID: 24719468

[B94] Li N. Han X. Feng D. Yuan D. Huang L.-J. (2019). Signaling crosstalk between salicylic acid and ethylene/jasmonate in plant defense: do we understand what they are whispering? Int. J. Mol. Sci. 20, 671. doi: 10.3390/ijms20030671, PMID: 30720746 PMC6387439

[B95] Li X. Heyer W.-D. (2008). Homologous recombination in DNA repair and DNA damage tolerance. Cell Res. 18, 99–113. doi: 10.1038/cr.2008.1, PMID: 18166982 PMC3087377

[B96] Li W. Huai X. Li P. Raza A. Mubarik M. S. Habib M.. (2021). Genome-wide characterization of glutathione peroxidase (GPX) gene family in rapeseed (Brassica napus L.) revealed their role in multiple abiotic stress response and hormone signaling. Antioxidants 10, 1481. doi: 10.3390/antiox10091481, PMID: 34573113 PMC8472808

[B97] Li P. Li X. Jiang M. (2021a). CRISPR/Cas9-mediated mutagenesis of *WRKY3* and *WRKY4* function decreases salt and Me-JA stress tolerance in *Arabidopsis thaliana* . Mol. Biol. Rep. 48, 5821–5832. doi: 10.1007/S11033-021-06541-4/FIGURES/6, PMID: 34351541

[B98] Li X. Sandgrind S. Moss O. Guan R. Ivarson E. Wang E. S.. (2021b). Efficient protoplast regeneration protocol and CRISPR/Cas9-mediated editing of glucosinolate transporter (GTR) genes in rapeseed (*Brassica napus* L.). Front. Plant Sci. 12, 680859. doi: 10.3389/fpls.2021.680859, PMID: 34305978 PMC8294089

[B99] Li T. Yang B. (2013). TAL effector nuclease (TALEN) engineering. Enzyme Engineering: Methods Protoc. 978, 63–72. doi: 10.1007/978-1-62703-293-3_5, PMID: 23423889

[B100] Li J. Yu X. Zhang C. Li N. Zhao J. (2022). The application of CRISPR/Cas technologies to Brassica crops: current progress and future perspectives. aBIOTECH 3, 146–161. doi: 10.1007/s42994-022-00076-3, PMID: 36304520 PMC9590542

[B101] Li L. Zhang D. Zhang Z. Zhang B. (2025). CRISPR/Cas: a powerful tool for designing and improving oil crops. Trends Biotechnol. 43, 773–789. doi: 10.1016/j.tibtech.2024.09.007, PMID: 39362812

[B102] Liu H. Ding Y. Zhou Y. Jin W. Xie K. Chen L.-L. (2017). CRISPR-P 2.0: an improved CRISPR-Cas9 tool for genome editing in plants. Mol. Plant 10, 530–532. doi: 10.1016/j.molp.2017.01.003, PMID: 28089950

[B103] Liu H. S. Li F. M. Xu H. (2004). Deficiency of water can enhance root respiration rate of drought sensitive but not drought-tolerant spring wheat. Agric. Water Manage. 64, 41–48. doi: 10.1016/s0378-3774(03)00143-4

[B104] Liu T. Liu Z. Song C. Hu Y. Han Z. She J. I.. (2012). Chitin-induced dimerization activates a plant immune receptor. Science 336, 1160–1164. doi: 10.1126/science.1218867, PMID: 22654057

[B105] Liu F. Selin C. Zou Z. Dilantha Fernando W. G. (2020). LmCBP1, a secreted chitin-binding protein, is required for the pathogenicity of *Leptosphaeria maculans* on *Brassica napus* . Fungal Genet. Biol. 136, 103320. doi: 10.1016/j.fgb.2019.103320, PMID: 31863838

[B106] Liu Y. Xin X. Li P. Wang W. Yu Y. Zhao X.. (2024). Editing of eIF (iso) 4E. c confers resistance against Turnip mosaic virus in Brassica rapa. Hortic. Plant J. 10, 1020–1034. doi: 10.1016/j.hpj.2024.05.001

[B107] Ma X. (2020). Highly efficient DNA-free plant genome editing using virally delivered CRISPR–Cas9. Nat. Plants. 6, 773–779. doi: 10.1038/s41477-020-0704-5, PMID: 32601419

[B108] Ma H. Kong C. Deng S. Zhao T. Ji J. Wang Y.. (2025). Resistance screening of cabbage to black rot and inheritance pattern analysis. Scientia Hortic. 345, 114129. doi: 10.1016/j.scienta.2025.114129

[B109] Ma C. Liu M. Li Q. Si J. Ren X. Song H. (2019). Efficient BoPDS gene editing in cabbage by the CRISPR/Cas9 system. Hortic. Plant J. 5, 164–169. doi: 10.1016/j.hpj.2019.04.001

[B110] Ma X. L. Zhang Q. Y. Zhu Q. L. Liu W. Chen Y. Qiu R.. (2015). A robust CRISPR/Cas9 system for convenient, high-efficiency multiplex genome editing in monocot and dicot plants. Mol. Plant 8, 1274–1284. doi: 10.1016/j.molp.2015.04.007, PMID: 25917172

[B111] Mahas A. Aman R. Mahfouz M. (2019). CRISPR-Cas13d mediates robust RNA virus interference in plants. Genome Biol. 20, 263. doi: 10.1186/s13059-019-1881-2, PMID: 31791381 PMC6886189

[B112] May D. Paldi K. Altpeter F. (2023). Targeted mutagenesis with sequence-specific nucleases for accelerated improvement of polyploid crops: Progress, challenges, and prospects. Plant Genome 16, e20298. doi: 10.1002/tpg2.20298, PMID: 36692095 PMC12806893

[B113] McDonald B. A. Linde C. (2002). Pathogen population genetics, evolutionary potential, and durable resistance. Annu. Rev. Phytopathol. 40, 349–379. doi: 10.1146/annurev.phyto.40.120501.101443, PMID: 12147764

[B114] Meena P. D. Mehta N. Saharan G. S. (2021). Minor pathogens: a worldwide challenge to cultivation of crucifers. Agric. Res. J. 58, 557–580. doi: 10.5958/2395-146x.2021.00081.8

[B115] Meng J.-Y. Liang G.-W. He Y.-J. Qian W. (2020). QTL mapping of salt and drought tolerance related traits in Brassica napus L. Acta Agron. Sin. (China) 47, 462–471. doi: 10.3724/SP.J.1006.2021.04034

[B116] Meraj T. A. Fu J. Raza M. A. Zhu C. Shen Q. Xu D.. (2020). Transcriptional factors regulate plant stress responses through mediating secondary metabolism. Genes 11, 346. doi: 10.3390/genes11040346, PMID: 32218164 PMC7230336

[B117] Merlot S. Leonhardt N. Fenzi F. Valon C. Costa M. Piette L.. (2007). Constitutive activation of a plasma membrane H+-ATPase prevents abscisic acid-mediated stomatal closure. EMBO J. 26, 3216–3226. doi: 10.1038/sj.emboj.7601750, PMID: 17557075 PMC1914098

[B118] Muhammad Aslam M. Waseem M. Jakada B. H. Okal E. J. Lei Z. Saqib H. S. A.. (2022). Mechanisms of abscisic acid-mediated drought stress responses in plants. Int. J. Mol. Sci. 23, 1084. doi: 10.3390/ijms23031084, PMID: 35163008 PMC8835272

[B119] Müller Paul H. Istanto D. D. Heldenbrand J. Hudson M. E. (2022). CROPSR: an automated platform for complex genome-wide CRISPR gRNA design and validation. BMC Bioinf. 23, 74. doi: 10.1186/s12859-022-04593-2, PMID: 35172714 PMC8848901

[B120] Murukarthick J. Sampath P. Lee S. C. Choi B.-S. Senthil N. Liu S.. (2014). BrassicaTED-a public database for utilization of miniature transposable elements in Brassica species. BMC Res. Notes 7, 1–11. doi: 10.1186/1756-0500-7-379, PMID: 24948109 PMC4077149

[B121] Muthusamy M. Son S. Park S. R. Lee S. I. (2023). Heat shock factor binding protein BrHSBP1 regulates seed and pod development in Brassica rapa. Front. Plant Sci. 14, 1232736. doi: 10.3389/fpls.2023.1232736, PMID: 37719218 PMC10499616

[B122] Nadeem M. Li J. Yahya M. Wang M. Ali A. Cheng A.. (2019). Grain legumes and fear of salt stress: focus on mechanisms and management strategies. Int. J. Mol. Sci. 20, 799. doi: 10.3390/ijms20040799, PMID: 30781763 PMC6412900

[B123] Nawaz G. Lee K. Park S. J. Kim Y. O. Kang H. (2018). A chloroplast-targeted cabbage DEAD-box RNA helicase BrRH22 confers abiotic stress tolerance to transgenic *Arabidopsis* plants by affecting translation of chloroplast transcripts. Plant Physiol. Biochem. 127, 336–342. doi: 10.1016/j.plaphy.2018.04.007, PMID: 29653436

[B124] Nellist C. F. Ohshima K. Ponz F. Walsh J. A. (2022). Turnip mosaic virus, a virus for all seasons. Ann. Appl. Biol. 180, 312–327. doi: 10.1111/aab.12755

[B125] Nerkar G. Devarumath S. Purankar M. Kumar A. Valarmathi R. Devarumath R.. (2022). Advances in crop breeding through precision genome editing. Front. Genet. 13. doi: 10.3389/fgene.2022.880195, PMID: 35910205 PMC9329802

[B126] Ng J. C. K. Perry K. L. (2004). Transmission of plant viruses by aphid vectors. Mol. Plant Pathol. 5, 505–511. doi: 10.1111/j.1364-3703.2004.00240.x, PMID: 20565624

[B127] Ngou B. P. M. Jones J. D. G. Ding P. (2022). Plant immune networks. Trends Plant Sci. 27, 255–273. doi: 10.1016/j.tplants.2021.08.012, PMID: 34548213

[B128] Nie X. Wang D. Pan Y. Hua Y. Lü P. Yang Y. (2024). Discovery, classification and application of the CPISPR-Cas13 system. Technol. Health Care 32, 525–544. doi: 10.3233/THC-230258, PMID: 37545273

[B129] Noman M. Aysha J. Ketehouli T. Yang J. Du L. Wang F.. (2021). Calmodulin binding transcription activators: An interplay between calcium signalling and plant stress tolerance. J. @ Plant Physiol. 256, 153327. doi: 10.1016/j.jplph.2020.153327, PMID: 33302232

[B130] Nuñez-Muñoz L. Vargas-Hernández B. Hinojosa-Moya J. Ruiz-Medrano R. Xoconostle-Cázares B. (2021). Plant drought tolerance provided through genome editing of the trehalase gene. Plant Signal. Behav. 16, 1877005. doi: 10.1080/15592324.2021.1877005, PMID: 33570447 PMC7971296

[B131] OECD-FAO (2023). OECD-FAO agricultural outlook 2023-2032 (Paris: OECD Publishing). doi: 10.1787/08801ab7-en

[B132] Okade H. Fujita Y. Miyamoto S. Tomoo K. Muto S. Miyoshi H.. (2009). Turnip mosaic virus genome-linked protein VPg binds C-terminal region of cap-bound initiation factor 4E orthologue without exhibiting host cellular specificity. J. Biochem. 145, 299–307. doi: 10.1093/jb/mvn180, PMID: 19122207 PMC4244113

[B133] Ordon J. Bressan M. Kretschmer C. Dall’Osto L. Marillonnet S. Bassi R.. (2020). Optimized Cas9 expression systems for highly efficient Arabidopsis genome editing facilitate isolation of complex alleles in a single generation. Funct. Integr. Genomics 20, 151–162. doi: 10.1007/s10142-019-00665-4, PMID: 30796544

[B134] Osakabe Y. Watanabe T. Sugano S. S. Ueta R. Ishihara R. Shinozaki K.. (2016). Optimization of CRISPR/Cas9 genome editing to modify abiotic stress responses in plants. Sci. Rep. 6, 26685. doi: 10.1038/srep26685, PMID: 27226176 PMC4880914

[B135] Pageau D. Lajeunesse J. Lafond J. (2006). Impact of clubroot [Plasmodiophora brassicae] on the yield and quality of canola. Can. J. Plant Pathol. 28 , 137–143.

[B136] Palukaitis P. Kim S. (2021). Resistance to turnip mosaic virus in the Family *Brassicaceae* . Plant Pathol. J. 37, 1–23. doi: 10.5423/PPJ.RW.09.2020.0178, PMID: 33551693 PMC7847761

[B137] Papikian A. Liu W. Gallego-Bartolomé J. Jacobsen S. E. (2019). Site-specific manipulation of Arabidopsis loci using CRISPR-Cas9 SunTag systems. Nat. Commun. 10, 729. doi: 10.1038/s41467-019-08736-7, PMID: 30760722 PMC6374409

[B138] Park J.-J. Dempewolf E. Zhang W. Wang Z.-Y. (2017a). RNA-guided transcriptional activation via CRISPR/dCas9 mimics overexpression phenotypes in Arabidopsis. PloS One 12, e0179410. doi: 10.1371/journal.pone.0179410, PMID: 28622347 PMC5473554

[B139] Park J. J. Dempewolf E. Zhang W. Wang Z. Y. (2017b). RNA-guided transcriptional activation via CRISPR/dCas9 mimics overexpression phenotypes in Arabidopsis. PloS One 12, pe0179410. doi: 10.1371/journal.pone.0179410, PMID: 28622347 PMC5473554

[B140] Pavlović I. Mlinarić S. Tarkowská D. Oklestkova J. Novák O. Lepeduš H.. (2019). Early Brassica crops responses to salinity stress: A comparative analysis between chinese cabbage, white cabbage, and kale. Front. Plant Sci. 10. doi: 10.3389/FPLS.2019.00450/BIBTEX, PMID: 31031786 PMC6470637

[B141] Podsędek A. (2007). Natural antioxidants and antioxidant capacity of Brassica vegeta bles: A review. LWT-Food Sci. Technol. 40, 1–11. doi: 10.1016/j.lwt.2005.07.023

[B142] Punja Z. K. Zhang Y. Y. (1993). Plant chitinases and their roles in resistance to fungal diseases. J. Nematol. 25, 526–540., PMID: 19279806 PMC2619419

[B143] Pyott D. E. Sheehan E. Molnar A. (2016). Engineering of CRISPR/Cas9-mediated potyvirus resistance in transgene-free Arabidopsis plants. Mol. Plant Pathol. 17, 1276–1288. doi: 10.1111/mpp.12417, PMID: 27103354 PMC5026172

[B144] Qu M. Havshøi N. W. Huang X. Shabala L. Yu M. Fuglsang A. T.. (2024). Understanding the mechanistic basis of ameliorative effects of boron on salinity in barley (*Hordeum vulgare*). Environ. Exp. Bot. 220, 105690. doi: 10.1016/J.ENVEXPBOT.2024.105690

[B145] Rahman H. Xu Y.-P. Zhang X.-R. Cai X.-Z. (2016). *Brassica napus* genome possesses extraordinary high number of CAMTA genes and CAMTA3 contributes to PAMP triggered immunity and resistance to *Sclerotinia sclerotiorum* . Front. Plant Sci. 7, 581. doi: 10.3389/fpls.2016.00581, PMID: 27200054 PMC4854897

[B146] Rahman M. U. Zulfiqar S. Raza M. A. Ahmad N. Zhang B. (2022). Engineering abiotic stress tolerance in crop plants through CRISPR genome editing. Cells. 11, 3590. doi: 10.3390/cells11223590, PMID: 36429019 PMC9688763

[B147] Raybould A. F. Maskell L. C. Edwards M.-L. Cooper J. I. Gray A. J. (1999). The prevalence and spatial distribution of viruses in natural populations of Brassica oleracea. New Phytol. 141, 265–275. doi: 10.1046/j.1469-8137.1999.00339.x, PMID: 33862926

[B148] Raza A. Razzaq A. Mehmood S. S. Hussain M. A. Wei S. He H.. (2021). Omics: The way forward to enhance abiotic stress tolerance in Brassica napus L. GM Crops Food 12, 251–281., PMID: 33464960 10.1080/21645698.2020.1859898 PMC7833762

[B149] Ren J. Petzoldt R. Dickson M. H. (2001). Screening and identification of resistance to bacterial soft rot in *Brassica rapa* . Euphytica 118, 271–280. doi: 10.1023/A:1017522501229

[B150] Rizhsky L. Liang H. Shuman J. Shulaev V. Davletova S. Mittler R. (2004). When defense pathways collide. The response of Arabidopsis to a combination of drought and heat stress. Plant Physiol. 134, 1683–1696. doi: 10.1104/pp.103.033431, PMID: 15047901 PMC419842

[B151] Robertson G. Burger J. Campa M. (2022). CRISPR/Cas-based tools for the targeted control of plant viruses. Mol. Plant Pathol. 23, 1701–1718. doi: 10.1111/mpp.13252, PMID: 35920132 PMC9562834

[B152] Roca Paixão J. F. Gillet F.-X. Ribeiro T. P. Bournaud C. Lourenço-Tessutti I. T. Noriega D. D.. (2019). Improved drought stress tolerance in *Arabidopsis* by CRISPR/dCas9 fusion with a histone acetyltransferase. Sci. Rep. 9, 8080. doi: 10.1038/s41598-019-44571-y, PMID: 31147630 PMC6542788

[B153] Romero M. Pérez M. (2024). Optimizing *Brassica oleracea* L. breeding through somatic hybridization using cytoplasmic male sterility (CMS) Lines: from protoplast isolation to plantlet regeneration. Plants 13, 3247. doi: 10.3390/plants13223247, PMID: 39599456 PMC11598112

[B154] Rusholme R. L. Higgins E. E. Walsh J. A. Lydiate D. J. (2007). Genetic control of broad-spectrum resistance to turnip mosaic virus in Brassica rapa (Chinese cabbage). J. Gen. Virol. 88, 3177–3186. doi: 10.1099/vir.0.83194-0, PMID: 17947545

[B155] Saha G. Park J.-I. Ahmed N. U. Kayum M. A. Kang K.-K. Nou I.-S. (2016). Characterization and expression profiling of MYB transcription factors against stresses and during male organ development in Chinese cabbage (Brassica rapa ssp. pekinensis). Plant Physiol. Biochem. 104, 200–215. doi: 10.1016/j.plaphy.2016.03.021, PMID: 27038155

[B156] Salehi-Lisar S. Y. Bakhshayeshan-Agdam H. (2016). Drought stress in plants: Causes, consequences, and tolerance. Drought Stress tolerance Plants (Cham) 1, 1–16. doi: 10.1007/978-3-319-28899-4_1

[B157] Sanfaçon H. (2015). Plant translation factors and virus resistance. Viruses 7, 3392–3419. doi: 10.3390/v7072778, PMID: 26114476 PMC4517107

[B158] Sato H. Mizoi J. Shinozaki K. Yamaguchi-Shinozaki K. (2024). Complex plant responses to drought and heat stress under climate change. Plant J. 117, 1873–1892. doi: 10.1111/tpj.16612, PMID: 38168757

[B159] Schaart J. G. van de Wiel C. C. M. Smulders M. J. M. (2021). Genome editing of polyploid crops: prospects, achievements and bottlenecks. Transgenic Res. 30, 337–351. doi: 10.1007/s11248-021-00251-0, PMID: 33846956 PMC8316217

[B160] Secchi M. A. Fernandez J. A. Stamm M. J. Durrett T. Prasad P. V. V. Messina C. D.. (2023). Effects of heat and drought on canola (*Brassica napus* L.) yield, oil, and protein: A meta-analysis. Field Crops Res. 293, 108848. do. doi: 10.1016/j.fcr.2023.108848

[B161] Sehgal A. Reddy K. R. Walne C. H. Barickman T. C. Brazel S. Chastain D.. (2022). Climate stressors on growth, yield, and functional biochemistry of two *Brassica* species, kale and mustard. Life (Basel) 12. doi: 10.1038/srep26685, PMID: 36294981 PMC9605623

[B162] Shah N. Anwar S. Xu J. Hou Z. Salah A. Khan S.. (2018). The response of transgenic Brassica species to salt stress: a review. Biotechnol. Lett. 40, 1159–1165. doi: 10.1007/S10529-018-2570-Z/TABLES/1, PMID: 29858710

[B163] Shahzad B. Rehman A. Tanveer M. Wang L. Park S. K. Ali A. (2022). Salt stress in Brassica: Effects, tolerance mechanisms, and management. J. Plant Growth Regul. 41, 781–795. doi: 10.1007/s00344-021-10338-x

[B164] Shihong Gao D. Zhu X. Lu B. (2021). Development and application of sensitive, specific, and rapid CRISPR-Cas13-based diagnosis. J. Med. Virol. 93, 4198–4204. doi: 10.1002/jmv.26889, PMID: 33599292 PMC8014745

[B165] Shopan J. Liu C. Hu Z. Zhang M. Yang J. (2020). Identification of eukaryotic translation initiation factors and the temperature-dependent nature of Turnip mosaic virus epidemics in allopolyploid *Brassica juncea* . 3 Biotech. 10, 75. doi: 10.1007/s13205-020-2058-0, PMID: 32051808 PMC6987279

[B166] Shopan J. Mou H. Zhang L. Zhang C. Ma W. Walsh J. A.. (2017). Eukaryotic translation initiation factor *2B-beta (eIF2Bβ)*, a new class of plant virus resistance gene. Plant J. 90, 929–940. doi: 10.1111/tpj.13519, PMID: 28244149

[B167] Soltabayeva A. Dauletova N. Serik S. Sandybek M. Omondi J. O. Kurmanbayeva A.. (2022). Receptor-like Kinases (LRR-RLKs) in response of plants to biotic and abiotic stresses. Plants (Basel) 11. doi: 10.3390/plants11192660, PMID: 36235526 PMC9572924

[B168] Soma F. Takahashi F. Yamaguchi-Shinozaki K. Shinozaki K. (2021). Cellular phosphorylation signaling and gene expression in drought stress responses: ABA-dependent and ABA-independent regulatory systems. Plants 10, 756. doi: 10.3390/plants10040756, PMID: 33924307 PMC8068880

[B169] Song Z.-X. Chu S.-J. Seo E.-Y. Hu W.-X. Lim Y. P. Park T.-S.. (2022). Construction of full-length infectious clones of turnip mosaic virus isolates infecting Perilla frutescens and genetic analysis of recently emerged strains in Korea. Arch. Virol. 167, 1089–1098. doi: 10.1007/s00705-021-05356-9, PMID: 35258649 PMC8902734

[B170] Spoel S. H. Dong X. (2012). How do plants achieve immunity? Defence without specialized immune cells. Nat. Rev. Immunol. 12, 89–100. doi: 10.1038/nri3141, PMID: 22273771

[B171] Stajič E. Kunej U. (2023). Optimization of cabbage (*Brassica oleracea* var. *capitata* L.) protoplast transformation for genome editing using CRISPR/Cas9. Front. Plant Sci. 14, 1245433. doi: 10.3389/fpls.2023.1245433, PMID: 37849838 PMC10577288

[B172] Stelmach-Wityk K. Szymonik K. Grzebelus E. Kiełkowska A. (2024). Development of an optimized protocol for protoplast-to-plant regeneration of selected varieties of *Brassica oleracea* L. BMC Plant Biol. 24, 1279. doi: 10.1186/s12870-024-06005-4, PMID: 39736572 PMC11686882

[B173] Sun Z. Li S. Chen W. Zhang J. Zhang L. Sun W.. (2021). Plant dehydrins: expression, regulatory networks, and protective roles in plants challenged by abiotic stress. Int. J. Mol. Sci. 22, 12619. doi: 10.3390/ijms222312619, PMID: 34884426 PMC8657568

[B174] Sun Q. Lin L. Liu D. Wu D. Fang Y. Wu J.. (2018). CRISPR/Cas9-mediated multiplex genome editing of the *BnWRKY11* and *BnWRKY70* genes in *Brassica napus* L. Int. J. Mol. Sci. 19, ), 2716. doi: 10.3390/ijms19092716, PMID: 30208656 PMC6163266

[B175] Takikawa Y. Takahashi F. (2014). Bacterial leaf spot and blight of crucifer plants (*Brassicaceae*) caused by *Pseudomonas syringae* pv. *maculicola* and *P. cannabina* pv. *alisalensis* . J. Gen. Plant Pathol. 80, 466–474. doi: 10.1007/s10327-014-0540-4

[B176] Talakayala A. Ankanagari S. Garladinne M. (2022). CRISPR-Cas genome editing system: a versatile tool for developing disease resistant crops. Plant Stress 3, 100056. doi: 10.1016/j.stress.2022.100056

[B177] Tan Z. Han X. Dai C. Lu S. He H. Yao X.. (2024). Functional genomics of Brassica napus: progress, challenges, and perspectives. J. Integr. Plant Biol. 66, 484–509. doi: 10.1111/jipb.13635, PMID: 38456625

[B178] Thomazella D. P. D. T. Seong K. Mackelprang R. Dahlbeck D. Geng Y. Gill U. S.. (2021). Loss of function of a DMR6 ortholog in tomato confers broad-spectrum disease resistance. Proc. Natl. Acad. Sci. U. S. A. 118, e2026152118. doi: 10.1073/pnas.2026152118, PMID: 34215692 PMC8271637

[B179] Ton L. (2024). Establishment of a CRISPR/CasRx system to modify Turnip mosaic virus (TuMV) resistance in *Brassica napus.* [dissertation]. [Perth, Western Australia]: The University of Western Australia.

[B180] Tsuda K. Katagiri F. (2010). Comparing signalling mechanisms engaged in pattern-triggered and effector-triggered immunity. Curr. Opin. Plant Biol. 13, 459–465. doi: 10.1016/j.pbi.2010.04.006, PMID: 20471306

[B181] Tu M. Wang R. Guo W. Xu S. Zhu Y. Dong J.. (2024). A CRISPR/Cas9-induced male-sterile line facilitating easy hybrid production in polyploid rapeseed (*Brassica napus*). Hortic. Res. 11, uhae139. doi: 10.1093/hr/uhae139, PMID: 38988621 PMC11233878

[B182] U, N (1935). Genome-analysis in Brassica with special reference to the experimental formation of *Brassica napus* and peculiar mode of fertilization. J. Jpn. Bot. 7, 389–452.

[B183] USDA (2023). European Union: oilseeds and products annual. Available online at: https://fas.usda.gov/data/european-union-oilseeds-and-products-annual-3 (Accessed October 20, 2024).

[B184] Van de Wouw A. P. Marcroft S. J. Ware A. Lindbeck K. Khangura R. Howlett B. J. (2014). Breakdown of resistance to the fungal disease, blackleg, is averted in commercial canola (*Brassica napus*) crops in Australia. Field Crops Res. 166, 144–151. doi: 10.1016/j.fcr.2014.06.023

[B185] Varanda C. M. R. Félix M. d. R. Campos M. D. Patanita M. Materatski P. (2021). Plant Viruses: from targets to tools for CRISPR. Viruses 13, 141. doi: 10.3390/v13010141, PMID: 33478128 PMC7835971

[B186] Vicente J. G. Holub E. B. (2013). *Xanthomonas campestris* pv. *Campestris* (cause of black rot of crucifers) in the genomic era is still a worldwide threat to brassica crops. Mol. Plant Pathol. 14, 2–18. doi: 10.1111/j.1364-3703.2012.00833.x, PMID: 23051837 PMC6638727

[B187] Villao-Uzho L. Chávez-Navarrete T. Pacheco-Coello R. Sánchez-Timm E. Santos-Ordóñez E. (2023). Plant promoters: their identification, characterization, and role in gene regulation. Genes 14, 1226. doi: 10.3390/genes14061226, PMID: 37372407 PMC10298551

[B188] Wada N. Osakabe K. Osakabe Y. (2022). Expanding the plant genome editing toolbox with recently developed CRISPR–Cas systems. Plant Physiol. 188, 1825–1837. doi: 10.1093/plphys/kiac027, PMID: 35099553 PMC8968252

[B189] Wahab A. Abdi G. Saleem M. H. Ali B. Ullah S. Shah W.. (2022). Plants’ physio-biochemical and phyto-hormonal responses to alleviate the adverse effects of drought stress: a comprehensive review. Plants (Basel) 11, 1620. doi: 10.3390/plants11131620, PMID: 35807572 PMC9269229

[B190] Walsh H. M. Rönkä A. Walsh J. A. (2023). Identification and genetic inheritance of a new source of broad-spectrum extreme resistance to turnip mosaic virus (TuMV) in *Brassica rapa* . Eur. J. Plant Pathol. 165, 693–699. doi: 10.1007/s10658-022-02634-3

[B191] Wang Z. Liu L. Cheng C. Ren Z. Xu S. Li X. (2020). GAI functions in the plant response to dehydration stress in *Arabidopsis thaliana* . Int. J. Mol. Sci. 21,819. doi: 10.3390/ijms21030819, PMID: 32012796 PMC7037545

[B192] Wang J. Mao L. Li Y. Lu K. Qu C. Tang Z.. (2024). Natural variation in *BnaA9.NF-YA7* contributes to drought tolerance in *Brassica napus* L. Nat. Commun. 15, 2082. doi: 10.1038/s41467-024-46271-2, PMID: 38453909 PMC10920887

[B193] Wang Z. P. Xing H. L. Dong L. Zhang H. Y. Han C. Y. Wang X. C.. (2015). Egg cell-specific promoter-controlled CRISPR/Cas9 efficiently generates homozygous mutants for multiple target genes in Arabidopsis in a single generation. Gen. Biol. 16, 144. doi: 10.1186/s13059-015-0715-0, PMID: 26193878 PMC4507317

[B194] Wang X. Yan B. Shi M. Zhou W. Zekria D. Wang H.. (2016). Overexpression of a *Brassica campestris* HSP70 in tobacco confers enhanced tolerance to heat stress. Protoplasma 253, 637–645. doi: 10.1007/s00709-015-0867-5, PMID: 26298102

[B195] Wani A. S. Ahmad A. Hayat S. Fariduddin Q. (2013). Salt-induced modulation in growth, photosynthesis and antioxidant system in two varieties of Brassica juncea. Saudi J. Biol. Sci. 20, 183–193. doi: 10.1016/j.sjbs.2013.01.006, PMID: 23961235 PMC3730539

[B196] Wei Y. S. Javed T. Liu T. T. Ali A. Gao S. J. (2025). Mechanisms of abscisic acid (ABA)-mediated plant defense responses: An updated review. Plant Stress 15, 100724. doi: 10.1016/j.stress.2024.100724

[B197] Wendel J. F. Lisch D. Hu G. Mason A. S. (2018). The long and short of doubling down: polyploidy, epigenetics, and the temporal dynamics of genome fractionation. Curr. Opin. Genet. Dev. 49, 1–7. doi: 10.1016/j.gde.2018.01.004, PMID: 29438956

[B198] Wu J. Cai G. Tu J. Li L. Liu S. Luo X.. (2013). Identification of QTLs for resistance to Sclerotinia stem rot and BnaC. IGMT5. a as a candidate gene of the major resistant QTL SRC6 in Brassica napus. PloS One 8, e67740. doi: 10.3389/fpls.2020.00577, PMID: 23844081 PMC3699613

[B199] Wu G. Fang X. Yu T. Chen J. Yan F. (2024). Turnip mosaic virus pathogenesis and host resistance mechanisms in *Brassica* . Hortic. Plant J. 10, 947–960. doi: 10.1016/j.hpj.2024.03.001

[B200] Wu J. Yan G. Duan Z. Wang Z. Kang C. Guo L.. (2020). Roles of the *Brassica napus* DELLA protein *BnaA6. RGA*, in modulating drought tolerance by interacting with the ABA signaling component BnaA10. ABF2. Front. Plant Sci. 11, 577., PMID: 32477388 10.3389/fpls.2020.00577 PMC7240051

[B201] Xu L. Lin Z. Tao Q. Liang M. Zhao G. Yin X.. (2014). Multiple NUCLEAR FACTOR Y transcription factors respond to abiotic stress in Brassica napus L. PloS One 9, e111354. doi: 10.1371/journal.pone.0111354, PMID: 25356551 PMC4214726

[B202] Xu Y. Zhan C. Huang B. (2011). Heat shock proteins in association with heat tolerance in grasses. Int. J. Proteom. 2011, 529648. doi: 10.1155/2011/529648, PMID: 22084689 PMC3200123

[B203] Yang J. Duan G. Li C. Liu L. Han G. Zhang Y.. (2019). The crosstalks between jasmonic acid and other plant hormone signaling highlight the involvement of jasmonic acid as a core component in plant response to biotic and abiotic stresses. Front. Plant Sci. 10, 1349. doi: 10.3389/fpls.2019.01349, PMID: 31681397 PMC6813250

[B204] Yang X. Lu M. Wang Y. Wang Y. Liu Z. Chen S. (2021). Response mechanism of plants to drought stress. Horticulturae 7, 50. doi: 10.3390/horticulturae7030050

[B205] Yang C. Wang E. Liu J. (2022). CERK1, more than a co-receptor in plant–microbe interactions. New Phytol. 234, 1606–1613. doi: 10.1111/nph.18074, PMID: 35297054

[B206] Yang H. Wu J.-J. Tang T. Liu K.-D. Dai C. (2017). CRISPR/Cas9-mediated genome editing efficiently creates specific mutations at multiple loci using one sgRNA in *Brassica napus* . Sci. Rep. 7 7489. doi: 10.1038/s41598-017-07871-9, PMID: 28790350 PMC5548805

[B207] Yang Y. Zhu K. Li H. Han S. Meng Q. Khan S. U.. (2018). Precise editing of *CLAVATA* genes in *Brassica napus* L. regulates multilocular silique development. Plant Biotechnol. J. 16, 1322–1335. doi: 10.1111/pbi.12872, PMID: 29250878 PMC5999189

[B208] Yao J. Bai J. Liu S. Fu J. Zhang Y. Luo T.. (2022). Editing of a novel Cd uptake-related gene *CUP1* contributes to reducing Cd accumulations in *Arabidopsis thaliana* and *Brassica napus* . Cells 11, 3888. doi: 10.3390/CELLS11233888/S1, PMID: 36497146 PMC9739810

[B209] Ye W. Hossain R. Pröbsting M. Ali A. A. M. Han L. Miao Y.. (2024). Knock-out of *BnHva22c* reduces the susceptibility of *Brassica napus* to infection with the fungal pathogen *Verticillium longisporum* . Crop J. 12, 503–514. doi: 10.1016/j.cj.2024.02.012

[B210] Yu E. Fan C. Yang Q. Li X. Wan B. Dong Y.. (2014). Identification of heat responsive genes in Brassica napus siliques at the seed-filling stage through transcriptional profiling. PloS One 9, e101914. doi: 10.1371/journal.pone.0101914, PMID: 25013950 PMC4094393

[B211] Yu F. Zhang X. Huang Z. Chu M. Song T. Falk K. C.. (2016). Identification of genome-wide variants and discovery of variants associated with Brassica rapa clubroot resistance gene Rcr1 through bulked segregant RNA sequencing. PloS One 11, e0153218., PMID: 27078023 10.1371/journal.pone.0153218 PMC4831815

[B212] Yu X. Yu J. Lu Y. Li W. Huo G. Zhang J.. (2024). An efficient and universal protoplast-based transient gene expression system for genome editing in Brassica crops. Hortic. Plant J. 10, 983–994. doi: 10.1016/j.hpj.2024.06.001

[B213] Zafar S. A. Zaidi S. Gaba Y. Singla-Pareek S. L. Dhankher O. P. Li X.. (2020). Engineering abiotic stress tolerance via CRISPR/Cas-mediated genome editing. J. Exp. Bot. 71, 470–479. doi: 10.1093/jxb/erz476, PMID: 31644801

[B214] Zahra N. Hafeez M. B. Kausar A. Al Zeidi M. Asekova S. Siddique K. H.. (2023). Plant photosynthetic responses under drought stress: Effects and management. J. Agron. Crop Sci. 209, 651–672. doi: 10.1111/jac.12652

[B215] Zaman Q. U. Chu W. Hao M. Shi Y. Sun M. Sang S.-F.. (2019). CRISPR/Cas9-mediated multiplex genome editing of JAGGED gene in Brassica napus L. Biomolecules 9, 725. doi: 10.3390/biom9110725, PMID: 31726660 PMC6921047

[B216] Zhan X. Liu W. Nie B. Zhang F. Zhang J. (2023). Cas13d-mediated multiplex RNA targeting confers a broad-spectrum resistance against RNA viruses in potato. Commun. Biol. 6, 855. doi: 10.1038/s42003-023-05205-2, PMID: 37591976 PMC10435558

[B217] Zhang K. He J. Liu L. Xie R. Qiu L. Li X.. (2020). A convenient, rapid and efficient method for establishing transgenic lines of Brassica napus. Plant Methods 16, 43. doi: 10.1186/s13007-020-00585-6, PMID: 32256679 PMC7106750

[B218] Zhang Y. Huang S. Wang X. Liu J. Guo X. Mu J.. (2018b). Defective APETALA2 genes lead to sepal modification in *Brassica* crops. Front. Plant Sci. 9, 367. doi: 10.3389/fpls.2018.00367, PMID: 29616073 PMC5869249

[B219] Zhang X. Li J. Cao Y. Huang J. Duan Q. (2023). Genome-wide identification and expression analysis under abiotic stress of *BrAHL* genes in *Brassica rapa* . Int. J. Mol. Sci. 24. doi: 10.3390/ijms241512447, PMID: 37569822 PMC10420281

[B220] Zhang Y. Liu J. Li Y. Ma H. Ji J. Wang Y.. (2025a). Generation of novel bpm6 and dmr6 mutants with broad-spectrum resistance using a modified CRISPR/Cas9 system in Brassica oleracea. J. Integr. Plant Biol. 67, 1214–1216. doi: 10.1111/jipb.13842, PMID: 39873367

[B221] Zhang K. Zhuo C. Wang Z. Liu F. Wen J. Yi B.. (2021). *BnaA03. WRKY28*, interacting with *BnaA09.VQ12*, acts as a brake factor of activated *BnWRKY33*-mediated resistance outburst against *Sclerotinia sclerotiorum* in *Brassica napus* . Plant 11, 609. doi: 10.3390/plants11050609, PMID: 35270079 PMC8912397

[B222] Zhang K. Liu F. Wang Z. Zhuo C. Hu K. Li X.. (2022). Transcription factor WRKY28 curbs WRKY33-mediated resistance to Sclerotinia sclerotiorum in Brassica napus. Plant Physiol. 190, 2757–2774. doi: 10.1093/plphys/kiac439, PMID: 36130294 PMC9706479

[B223] Zhang Y. Massel K. Godwin I. D. Gao C. (2018). Applications and potential of genome editing in crop improvement. Genome Biol. 19, 210. doi: 10.1186/s13059-018-1586-y, PMID: 30501614 PMC6267055

[B224] Zhang L. Meng S. Liu Y. Han F. Xu T. Zhao Z.. (2024). Advances in and perspectives on transgenic technology and CRISPR-Cas9 gene editing in broccoli. Genes 15, 668. doi: 10.3390/genes15060668, PMID: 38927604 PMC11203320

[B225] Zhang K. Nie L. Cheng Q. Yin Y. Chen K. Qi K. (2019). Effective editing for lysophosphatidic acid acyltransferase 2/5 in allotetraploid rapeseed (*Brassica napus* L.) using CRISPR-Cas9 system. Biotechnol. Biofuels 12, 225. doi: 10.1186/s13068-019-1567-8, PMID: 31548867 PMC6753616

[B226] Zhang Y. Wang R. Luo T. Fu J. Yin M. Wang M.. (2025b). CRISPR-mediated *BnaNRAMP1* homologous copies editing create a low Cd-accumulation oilseed rape germplasm with unaffected yield. J. Integr. Agric. 24, 1704–1717. doi: 10.1016/J.JIA.2024.05.016

[B227] Zhang R. Zhang C. Lyu S. Wu H. Yuan M. Fang Z.. (2022). BcTFIIIA Negatively regulates turnip mosaic virus infection through interaction with viral CP and VPg proteins in pak choi (*Brassica campestris* ssp. *chinensis*). Genes (Basel) 13. doi: 10.3390/genes13071209, PMID: 35885992 PMC9317785

[B228] Zhang D. Zhang Z. Unver T. Zhang B. (2020). CRISPR/Cas: A powerful tool for gene function study and crop improvement. J. Adv. Res. 29, 207–221. doi: 10.1016/j.jare.2020.10.003, PMID: 33842017 PMC8020163

[B229] Zhang T. Zheng Q. Yi X. An H. Zhao Y. Ma S.. (2018a). Establishing RNA virus resistance in plants by harnessing CRISPR immune system. Plant Biotechnol. J. 16, 1415–1423. doi: 10.1111/pbi.12881, PMID: 29327438 PMC6041442

[B230] Zhao D. Xia X. Su J. Wei M. Wu Y. Tao J. (2019). Overexpression of herbaceous peony HSP70 confers high temperature tolerance. BMC Genome 20, 70. doi: 10.1186/s12864-019-5448-0, PMID: 30665351 PMC6341652

[B231] Zhao C. Zhang Y. Gao L. Xie M. Zhang X. Zeng L.. (2024). Genome editing of *RECEPTOR-LIKE KINASE* 902 confers resistance to necrotrophic fungal pathogens in *Brassica napus* without growth penalties. Plant Biotechnol. J. 22, 538–540. doi: 10.1111/pbi.14253, PMID: 38047432 PMC10893935

[B232] Zheng X. Koopmann B. Ulber B. von Tiedemann A. (2020a). A global survey on diseases and pests in oilseed rape-current challenges and innovative strategies of control. Front. Agron. 2, 590908. doi: 10.3389/fagro.2020.590908

[B233] Zheng M. Zhang L. Tang M. Liu J. Liu H. Yang H.. (2020b). Knockout of two *Bna MAX* 1 homologs by CRISPR/Cas9-targeted mutagenesis improves plant architecture and increases yield in rapeseed (*Brassica napus* L.). Plant Biotechnol. J. 18, 644–654., PMID: 31373135 10.1111/pbi.13228 PMC7004912

[B234] Zhou X. Zhong T. Wu M. Li Q. Yu W. Gan L.. (2024). Multiomics analysis of a resistant European turnip ECD04 during clubroot infection reveals key hub genes underlying resistance mechanism. Front. Plant Sci. 15, 1396602. doi: 10.3389/fpls.2024.1396602, PMID: 38845850 PMC11153729

[B235] Zhu M. Lu S. Zhuang M. Zhang Y. Lv H. Ji J.. (2021). Genome-wide identification and expression analysis of the *Brassica oleracea* L. chitin-binding genes and response to pathogens infections. Planta 253, 80. doi: 10.1007/s00425-021-03596-2, PMID: 33742226 PMC7979657

